# Inferring clonal somatic mutations directed by X chromosome inactivation status in single cells

**DOI:** 10.1186/s13059-024-03360-1

**Published:** 2024-08-09

**Authors:** Ilke Demirci, Anton J. M. Larsson, Xinsong Chen, Johan Hartman, Rickard Sandberg, Jonas Frisén

**Affiliations:** 1 Department of Cell and Molecular Biology, Karolinska Institutet, Stockholm, Sweden; 2 Department of Oncology-Pathology, Karolinska Institutet, Stockholm, Sweden; 3 Department of Clinical Pathology and Cancer Diagnostics, Karolinska University Hospital, Stockholm, Sweden

**Keywords:** Mutations, Clonality, X chromosome inactivation, Hematopoiesis

## Abstract

Analysis of clonal dynamics in human tissues is enabled by somatic genetic variation. Here, we show that analysis of mitochondrial mutations in single cells is dramatically improved in females when using X chromosome inactivation to select informative clonal mutations. Applying this strategy to human peripheral mononuclear blood cells reveals clonal structures within T cells that otherwise are blurred by non-informative mutations, including the separation of gamma-delta T cells, suggesting this approach can be used to decipher clonal dynamics of cells in human tissues.

**Supplementary Information:**

The online version contains supplementary material available at 10.1186/s13059-024-03360-1.

## Background

**Fig. 1:**
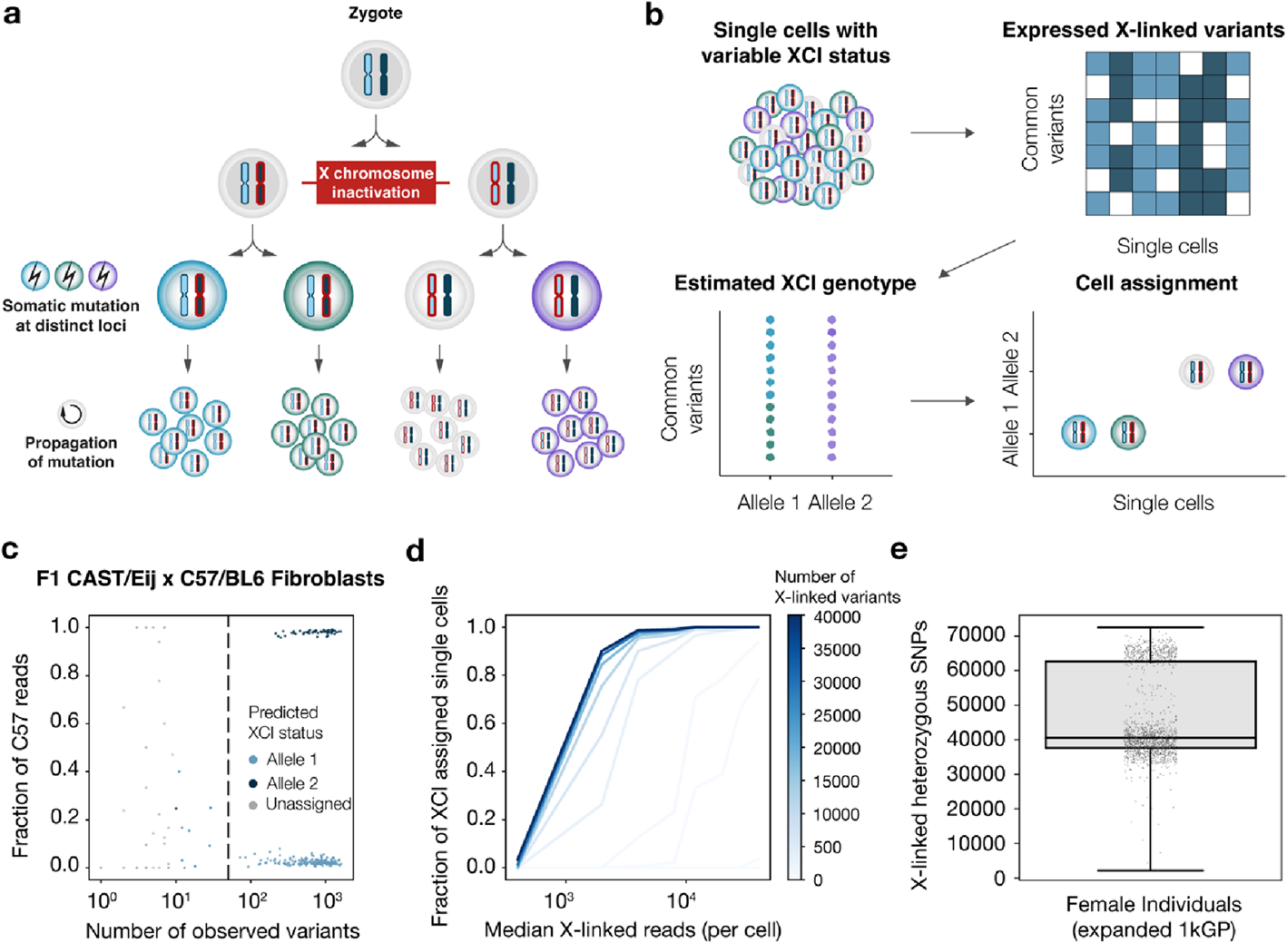
Assignment of active X chromosome alleles to single cells in females. **a** During early development in female therian mammals, one of the two X chromosomes is randomly inactivated in a heritable manner. Somatic mutations which occur after X chromosome inactivation are therefore associated with an active X allele. Larger X chromosomes reflect active alleles. Colors denote nuclear or mitochondrial mutations. **b** Since most X-linked transcripts are produced by the active X allele, expressed single-nucleotide polymorphisms can be utilized to infer X chromosome haplotypes and assign each single cell to a predicted XCI status based on these haplotypes. **c** Validation of the XCI deconvolution approach. A scatter plot of the number of observed X-linked variants compared to the fraction of C57 allelic reads (paternal allele), where each cell is colored by its predicted XCI status. Dashed vertical line is a quality cutoff (*n* = 50 observed variants). *n* = 53, 188 and 34 allele 1, allele 2 and unassigned cells without filtering. *n* = 52 and 180 allele 1 and allele 2 after filtering (dashed line). After filtering, estimated XCI and actual XCI based on the fraction of C57 reads agree 100%. **d** Fraction of XCI assigned quality filtered cells (*n* = 232 cells) as a function of number of X-linked reads (*x*-axis) and the number of X-linked variants (color). **e** Number of X-linked heterozygous SNPs found in genic regions in human females (*n* = 1604 individuals). Center lines denote the median; hinges denote the first and third quartiles; whiskers denote 1.5 × the interquartile range (IQR)

It has recently been demonstrated that lineage relationships can be assessed at the single cell level based on mitochondrial mutations harbored by cells which share a common ancestor [1, 2]. However, these putative lineage markers may often be affected by technical variation, or homoplasy, and often rely on subjective selection of informative mutations. Here, we reasoned that X chromosome inactivation (XCI) status of cells from female donors could be used to resolve clonal structures based on mutations that propagate along with the active X allele to daughter cells as stable tags (Fig. 1a). XCI is established in the epiblast of the post-implantation embryo in humans, resulting in mosaicism of maternal and paternal active X alleles in somatic tissue of XX females [3, 4]. Following gastrulation, cells forming the three germ layers engage in rapid proliferation where somatic mutation occurs during cell division [5], which are consequently passed on to daughter cells together with a stable active X allele thereafter.

## Results

We first sought to demonstrate how the XCI status of single female cells can be determined by using a Bayesian XCI deconvolution approach for predicting the active X chromosome in single cells from single-cell RNA sequencing (scRNA-seq) data (the  “Methods” section) [6, 7]. We utilized the demultiplexing tool *Vireo* to determine XCI status [6]. By restricting the variants used for the deconvolution to the X chromosome and specifying two expected donors, the *Vireo* algorithm effectively detects the paternal and maternal allele. The expressed paternal and maternal variants can then be used to assign XCI status to each cell (Fig. 1b). To validate our approach, we applied the XCI deconvolution approach to a set of mouse primary fibroblast cells from a female F1 mouse of a CAST/EiJ x C57/BL6J cross. The genetic variation between the two strains allows each cell to be assigned to the paternal or maternal allele based on expressed X-linked variants (*n* = 52 and 180 CAST/EiJ and C57/BL6J active-X cells, respectively). The XCI deconvolution approach completely agreed with the ground truth, confirming the accuracy of our approach (Fig. 1c). To investigate the sequencing depth and single nucleotide polymorphism (SNP) density required to confidently assign cells to their XCI status, we downsampled both the number of sequenced reads and the variants used to predict XCI status (Additional File 1: Fig. S1a). The approach maintains the high recovery of XCI status for all high-quality cells down to 3000 X-linked reads (120,000 reads total, Additional File 1: Fig. S1b) and 12,000 heterozygous genic SNPs, after which the performance becomes worse (Fig. 1d). Moreover, we found that in all cases the XCI status was either correctly assigned or left unassigned (Additional File 1: Fig. S2). Importantly, by analyzing X-linked variants of females included in the expanded 1000 genomes, we found that the number of heterozygous SNPs found on the X chromosome in female humans is well above the required variation across the human population (Fig. 1e, Additional File 1: Fig. S3). Moreover, a previous study found that only a very small fraction of the female population has a heavily skewed XCI ratio, with a mean ratio 48:52 in adult females [8] Therefore, the XCI deconvolution approach is widely applicable to human cells from any female donor.

**Fig. 2:**
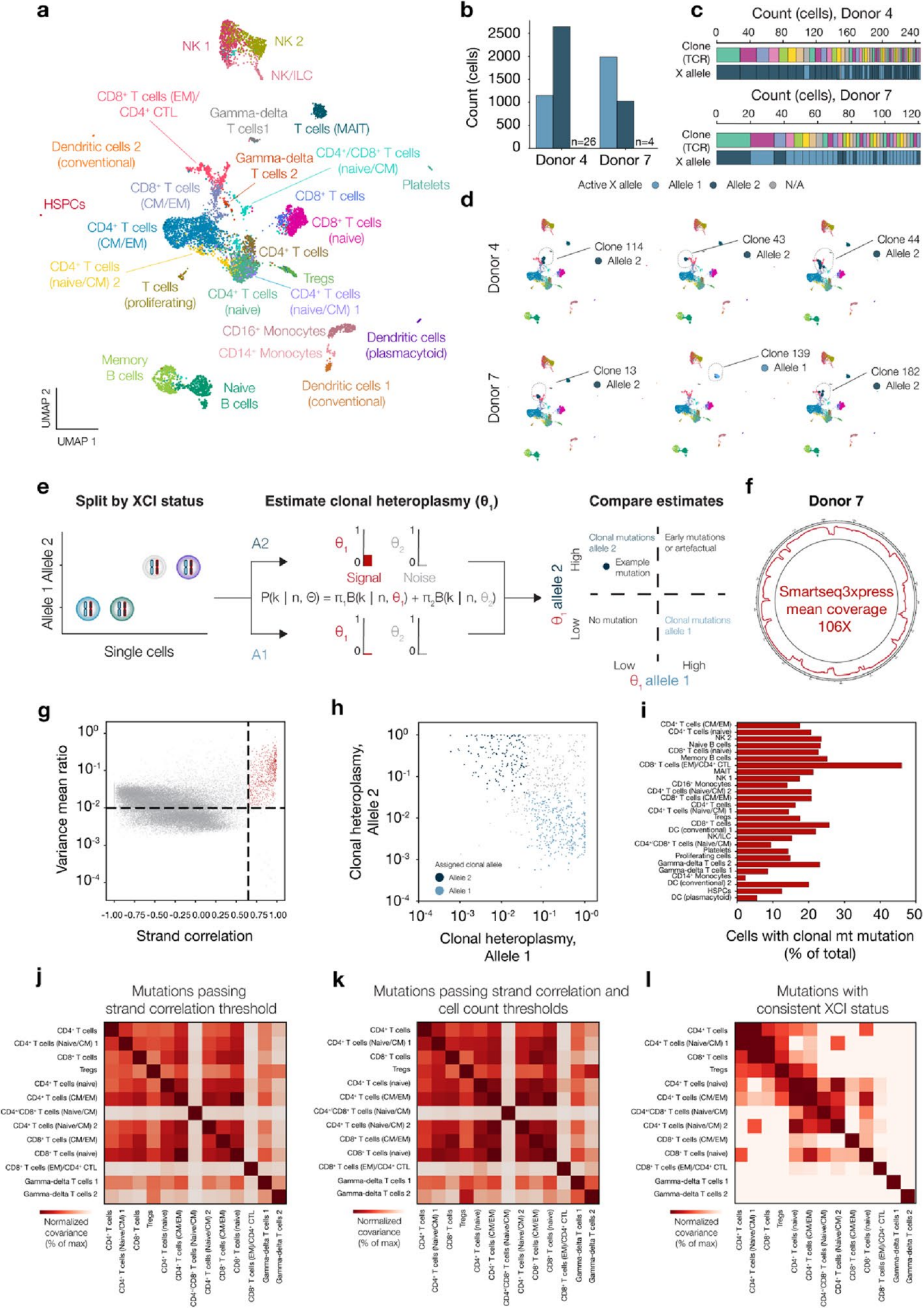
Application of XCI status deconvolution for clonal mitochondrial mutation discovery in human PBMCs. **a** Visualization of 6837 PBMCs from two female donors (donor 4 *n* = 3,820 and donor 7 *n* = 3,017) with UMAP. HSPC, hematopoietic stem and progenitor cell; CM, central memory; EM, effector memory; CTL, cytotoxic T lymphocyte; MAIT, mucosal associated invariant T cell; NK, natural killer cell; ILC, innate lymphoid cell. Populations annotated as CD4^+^ T cells and CD8^+^ T cells are T cells which clustered by their specific TCR gene expression. **b** Deconvolution of active X-alleles and assignment of XCI status to single cells in two blood donors. For donor 4, allele 1 *n* = 1,153, allele 2 *n* = 2,641, and unassigned *n* = 26 cells. For donor 7, allele 1 *n* = 1,988, allele 2 *n* = 1,025, and unassigned *n* = 4 cells. **c** Clonality of cells based on TCR sequence compared to XCI status, for donor 4 and donor 7. Colors denote individual T cell clones. Each pair of stacked bars corresponds to a single cell. **d** Visualization of the three most abundant TCR clones with UMAP, for both donors (top: donor 4, bottom: donor 7). Each cell is colored by XCI status. **e** Schematic of computational strategy to identify clonal mitochondrial mutations based on XCI status of single cells. **f** Coverage over the mitochondrial genome based on full-length transcriptomes produced with Smartseq3xpress for PBMCs from donor 7. **g** Variance mean ratio of variant detection compared to strand correlation, *n* = 40,998 total variants and *n* = 766 variants passing quality threshold for donor 7. **h** Estimated heteroplasmy of cells harboring threshold passing mitochondrial mutations separated by XCI status. **j** Percentage of PBMCs with clonal mitochondrial mutations for each cell type from donor 7. **i** Fate coupling of T cells based on normalized covariance of mutation counts passing strand correlation threshold for heteroplasmy. **k** Fate coupling of T cells based on normalized covariance of mutation counts which maintain the heteroplasmy criteria and present in more than 5 cells and less than 500 cells. **l** T cell lineage reconstruction based on clonal mitochondrial mutations for donor 7. The scale denotes normalized covariance of clonal mitochondrial mutations across cell types

To evaluate the use of XCI status and to confirm measured somatic variation as clonal markers, we reanalyzed human peripheral blood mononuclear cells (PBMCs) from female donors sequenced with Smart-seq3xpress [9]. Dimensionality reduction of all transcriptomes showed 27 clusters where all main cell types in the human PBMCs were represented in both donors (Fig. 2a). More than 99% of cells from the two female donors, donor 4 and donor 7, could be assigned an XCI status (Fig. 2b). Most cell clusters followed the XCI bias that was observed in the overall cell population, although a few cell clusters were inconsistent with the overall bias, suggesting exceptionally large clones present in those clusters (e.g., “CD8 + T cells (EM)/CD4 + CTL” in donor 4, “CD8 + T cells (EM)/CD4 + CTL” and “Memory B cells” in donor 7, Additional File 1: Fig. S4a). Analyzing PBMCs enabled us to also use clonal immune receptors [10] information as independent clonal information, and all clonal T cell receptor (TCR) sequences agreed with XCI status (Fig. 2c, 246 cells across 52 clones for donor 4 and 121 cells across 35 clones for donor 7), except for clone 386 from donor 4, which likely was due to incorrect calling based on poor data quality for this clone. In contrast, shuffling the XCI status of all cells resulted in no clones with consistent XCI status, except for small clones where all cells were assigned the same XCI status by chance (Additional File 1: Fig. S4b-c). Further visualization of the most abundant T cell clones and their respective XCI status showed that effector memory CD8^+^ T cell population shows the highest level of clonal expansion in both donors and mucosal-associated T cells in donor 7 (Fig. 2d). Therefore, the successful assignment of XCI status to T cell clones shows that the XCI deconvolution was accurate and demonstrates its usefulness as a necessary condition to classify somatic variation as a clonal marker.

We then developed a statistical approach to use XCI status to select informative clonal mitochondrial mutations from an identified set of putatively somatic mutations, based on splitting cells according to XCI status and calling mitochondrial mutations separately using a binomial mixture model (Fig. 2e, the “Methods” section). In addition to mutation selection, our approach uses the binomial mixture model to assign posterior probabilities of mutation presence in single cells, in contrast to the previously described software *Mquad* which relies on a similar statistical model only for mutation selection [11]. We assessed our approach to detect clonal mutations using a simulation approach which modeled many possible factors that might impact sensitivity of detection. The factors included XCI ratio, number of cells profiled, the fraction of cells in the population with the mutation, heteroplasmy level of the mutation, and the number of sequencing reads covering the variant. The main factors that impacted sensitivity were heteroplasmy level and read depth. XCI ratio did not have an impact on sensitivity. At the read depths commonly used for mitochondrial mutation discovery (100X coverage), our approach is generally able to detect clonal mutations at a heteroplasmy level of 4% and above (Additional File 1: Fig. S5). Moreover, we also assessed the sensitivity and specificity of mutation calling in single cells using the binomial mixture model, and we found excellent sensitivity at high specificity at heteroplasmy level 4% and above (Additional File 1: Fig. S6).

We focused on donor 7 due to the high mitochondrial genome coverage (106X, Fig. 2f), compared to donor 4 (49X, data not shown). We obtained the initial set of mitochondrial mutations using the *mgatk* software [2], established quality filtering thresholds, and identified 766 putative somatic mitochondrial mutations (Fig. 2g), and after discarding mutations where neither allele passed the clonal heteroplasmy threshold, 689 mutations remained (the “Methods” section). Selecting clonal mutations based on XCI status validated 480 of these mutations (Fig. 2h), hereafter called clonal mitochondrial mutations, whereas the remaining ~ 30% of mutations did not have a consistent XCI status and were discarded. The XCI status of the groups of cells harboring discarded mutations was consistent with randomly sampling cells from the whole dataset (Additional File 1: Fig. S7). We also compared this set of mutations to the mutations obtained by *Mquad*. *Mquad* identified 310 variants using an approach orthogonal to *mgatk* based on model-based identification using the binomial mixture model. Reassuringly, the heteroplasmy levels of mutations found by *Mquad* highly agreed with the maximum heteroplasmy level across alleles found by our approach (Additional File 1: Fig. S8a). By computing the logit difference between the inferred heteroplasmy of the two alleles, we found that many of the variants found by *Mquad* had similar heteroplasmy levels on both regardless of XCI status, and 82 variants remained after this filtering based on this metric. Interestingly, this procedure removed all the variants found in many cells in the population (Additional File 1: Fig. S8b). Out of the 82 mutations, 35 were only found by *Mquad*, and 16 of these mutations passed the same criteria we established for *mgatk.* These variants generally had high strand correlation, which was slightly below the 0.65 cut-off, one of the quality criteria used by *mgatk* to identify variants (Additional File 1: Fig. S8c). Overall, the *Mquad and mgatk* discovered a surprising number of different variants, although most variants discovered only by *Mquad* did not pass our filtering (Additional File 1: Fig. S8d).

Due to the higher number of variants discovered by *mgatk*, we continued with this set of mutations for further analysis. Twenty percent of all cells harbored at least one clonal mitochondrial mutation, largely irrespective of cell type (Fig. 2i). CD8^+^ effector memory T cells/CD4^+^ cytotoxic T lymphocytes were an exception with approximately 50% of cells harboring a clonal mitochondrial mutation, although these populations of cells were less abundant in the whole dataset (Fig. 2a, 2i, Additional File 1: Fig. S9a). Overall, a higher number of clonal mitochondrial mutations were detected in the CD8^+^ T cell population which clustered only based on their specific expression of TCR genes compared to their CD4^+^ counterparts (Additional File 1: Fig. S9b). Three of the mitochondrial mutations marked four of the T cell clones, with 100% concordance (2860G > A found in clone 130 and 445, 6253 T > C found in clone 13, and 6793 T > C found in clone 406) (Additional File 1: Fig. S10). Confident that these mitochondrial mutations mark closely related cells, we investigated the fate coupling of T cell lineages based on normalized covariance of clonal mitochondrial mutations present in more than one cell, with a comparison to previous approaches (Fig. 2j–l). Several models have been proposed for T cell development, where, e.g., the lineage relationship between memory and effector T cells remains unclear [12]. Furthermore, while the gamma-delta subset of T cells is phenotypically different from alpha–beta T cells (CD4 + and CD8 +), it is unclear which thymic progenitors produce gamma-delta T cells and the extent to which they share progenitors with alpha–beta T cells [13, 14]. In mice, the timing alpha–beta and gamma-delta T cell fate decisions are quite well characterized [15]. However, the development of human gamma-delta T cells is less clear [16]. Understanding the origin of gamma-delta T cells may have implications for their role in tumor immune surveillance [17]. Our fate coupling analysis based on clonal mitochondrial mutations obtained higher resolution than using mitochondrial mutations without XCI-informed selection (Fig. 2j). The lineage structure could not be resolved even when including only the mitochondrial mutations present in more than 5 cells and less than 500 cells in our analysis, which is a previously established cutoff [2] for selecting informative mutations (Fig. 2k). We observed that CD4^+^ and CD8^+^ T cell compartments overall share clonal mutations, suggesting a common lymphoid ancestor, as expected [18, 19] (Fig. 2l). However, naïve CD4^+^ T cells were not coupled with a subset of CD4^+^ CM T cells. In contrast, naïve CD8^+^ T cells shared clonal mutations across all CD8^+^ T cell populations, suggesting that there are distinct routes of lineage restriction shaping the CD4^+^ and CD8^+^ T cell hierarchies. Finally, we found that gamma-delta T cells do not share mutations with other T cell lineages. Although the gamma-delta T cells clusters were coupled without XCI-informed selection of variants, they shared many mutations with other T cell lineages that were removed by the XCI-informed selection step, suggesting these subsets are produced by a separate set of thymic progenitors than CD4^+^ and CD8^+^ T cells.

To demonstrate the wide applicability of our approach, we applied the XCI deconvolution to 10× ATAC-seq with enrichment for the mitochondrial genome (mtscATAC-seq) from a colorectal cancer sample [2] (Additional File 1: Fig. S11a). The chromatin of the inactive X allele is condensed after inactivation [20], rendering it inaccessible by assays that capture DNA in open chromatin regions such as ATAC-seq. Therefore, single-cell chromatin accessibility measurements can be used in lieu of scRNA-seq for XCI status deconvolution. All cell clusters showed heterogeneous XCI status, including the epithelial cell cluster (Additional File 1: Fig. S11b). The mitochondrial mutations reported in the original manuscript were either present in the epithelial or immune cell population (Additional File 1: Fig. S11c). Out of the 12 mitochondrial mutations which were initially reported, 4 mutations had consistent XCI status. Interestingly, while most of the mitochondrial mutations reported in the epithelial cells were of mixed XCI status (16147C > T, 12889G > A, 9728C > T, 1227G > A, 6081G > A), many of the mutations detected in the immune cells had one consistent X allele active (9804G > A, 3244G > A, 12731 T > C) (Additional File 1: Fig. S12). This finding suggests that some of the reported mitochondrial mutations in the epithelial cell cluster are not reflective of the subclonal structure of the tumor, whereas the mutations found in the immune cells are reflective of the clonal relationships. Therefore, the XCI status can be applied to scATAC-seq data as evidenced by the mitochondrial mutations detected in the immune cells. We further applied the XCI inference to a 10× scRNA-seq dataset of B and T cells from a breast tumor, with targeted VDJ sequencing [21] (Additional File 1: Fig. S13a). Each clone as defined by its immune receptor sequence had a consistent XCI status with few exceptions (Additional File 1: Fig. S13b-c), particularly when compared to a shuffled control (Additional File 1: Fig. S13d-e). The two largest B cell clones were plasma cells with allele 1 active, while most of the large T cell clones had allele 2 active and were spread out in the T cell cluster (Additional File 1: Fig. S13d and S13f). Therefore, the approach can be applied to different data modalities and is agnostic to the used library preparation method. Taken together, selection of clonal mutations is a crucial step for the analysis of lineage relationships in humans, and the X chromosome inactivation status of single cells facilitates the discrimination of uninformative mutations when applied to data generated by a wide variety of available methods.

Apart from the mitochondrial genome, the nuclear genome may contain somatic single nucleotide variants (SNVs) that are informative of clonal relationships. To demonstrate that our approach can be applied to nuclear SNVs, we applied the recently published variant calling software *Monopogen* to the donor 7 PBMCs [22]. After the application of hard filtering steps used by *Monopogen*, we detected 1153 putatively somatic nuclear SNVs. Most of these SNVs were detected in 2–10 cells, which is consistent with the clonal diversity in the PBMC dataset we analyzed (Additional File 1: Fig. S14a). Considering XCI status of the cells harboring these putative SNVs, we filtered away 759 putative SNVs with inconsistent XCI status, with 394 mutations remaining (333 with allele 1 active and 61 with allele 2 active). The SNVs present in a relatively high number of cells all had inconsistent XCI status (Additional File 1: Fig. S14b). The majority of these mutations were found in 2 cells, with a few mutations found in larger groups of cells (Additional File 1: Fig. S14c-e). About 20% of cells had at least one clonal nuclear somatic mutation detected across cell clusters (Additional File 1: Fig. S14f). These results demonstrate the use of XCI status to select clonally informative nuclear somatic SNVs, although further research is needed to account for the significantly lower variant coverage and variation in gene expression across cell types which may distort inferred lineage relationships.

## Discussion

Here, we demonstrated that using XCI status is an effective strategy to select clonally informative mutations for analyses that effectively removes more widespread mutations or artefactual observations. The use of somatic variation to assign clonal relationships have a clear trade-off between precision and recall. Current methods to detect and assign somatic mutation events as marking related cells have limited ways to confirm that the discovered mutations occurred during an appropriate timeframe for clonal analysis. The approach introduced here significantly increases the precision in the sense that the selected mutations are guaranteed to have been acquired at a later point in development. One clear limitation of this approach is the inability to identify of sub-clonal relationships between cells which have undergone a bottleneck event after XCI. For example, identifying sub-clonal mutations in B or T cells will not be helped by the XCI approach, since these cells all already share XCI status. Another example would be identifying clonal mutations within cancers which originate from a single cell. The discarded mutations which are detected in cells with different XCI status may be discarded due to several different reasons. First, the mutation may be due to homoplasy in the individual, which we cannot completely rule out if the mutation occurs multiple times in different cells with the same XCI status. Second, the mutation may have occurred before XCI and should therefore be detected in most cells in the population. Third, the mutation may be artefactual, either due to errors introduced during library preparation or due to mapping errors during analysis. Understanding which mutations are artefactual as well as which are preceding XCI or homoplastic may further improve the sensitivity and specificity of lineage analysis and is subject to future work. While this study mainly focused on scRNA-seq for XCI status deconvolution and mitochondrial mutation calling, we have also demonstrated its applicability in droplet-based scATAC-seq, scRNA-seq of nuclear mutations and scRNA-seq with targeted enrichment of somatic variation.

## Conclusions

In conclusion, using XCI status to classify clonal somatic mutations is an accessible and accurate approach that will contribute to our understanding of human development under physiological conditions.

## Methods

### Data processing and allelic read counting of mouse fibroblasts

The mouse fibroblast data was processed as previously described [23]. Briefly, the data was processed using zUMIs (v2.4.1) with STAR (v2.5.4b) to the mm10 genome with CAST variants N-masked to prevent mapping bias. The allelic read counting was based on a filtered set of variants also described in the same publication. For each cell, the number of reads supporting the C57 and CAST alleles based on variant support was counted. Downsampling of reads was done with *samtools* (v1.7, *htslib* v1.9) and downsampling of variants was done with *bcftools* (v1.16, *htslib* 1.16) and *vcflib* (v1.0.0).

### Estimating XCI status by genotype deconvolution

X-linked variants were counted per cell using *cellsnp-lite* (v1.2.2, *htslib* v1.14) using the SNPs found by the 1000 genomes project [24], with minimum allele frequency 0.01 and minimum variant count 5. Then, *vireo* (v0.5.7) was applied to the cells with 2 expected donors.

### Counting heterozygous X-linked variants in humans

To count the number of heterozygous X-linked variants present in genic regions, the vcf file from the female individuals from the extended 1000 genomes project was analyzed. To select variants which are only present in genic regions, the gene coordinates of human genes from Ensembl (Ensembl Human genes 108, GRCh38.p13) was intersected with the X-linked variants. Then, the Python package *scikit-allele* (v1.3.2) was used to count heterozygous variants for each individual based on the filtered vcf file.

### Analysis of human PBMCs

The dataset of human PBMCs was obtained from Hagemann-Jensen et al. [9], including a subset for the two female donors, donor 4 and donor 7. To reproduce the dimensionality reduction representation of the cells, the data was filtered, normalized, and integrated exactly as described in Hagemann-Jensen et al. The cell type annotations for each cell were kept as previously published, except for “Clonal CD4 + T cells” and “Clonal CD8 + T cells” which were renamed “CD4 + T cells” and “CD8 + T cells” respectively. TCR reconstruction was performed as described in Hagemann-Jensen et. al. by using TraCeR [25]. To define clones, the maximum of VJ and VDJ sequence distance was used to cluster cells by Leiden clustering using Scirpy. If there were multiple immune receptors detected, the minimum distance was used. Each connected module as defined by the Leiden clustering was then considered a clone.

### Mitochondrial mutation calling assisted by XCI status

Let $k={k}_{1},{k}_{2},\dots ,{k}_{S}$ be the set of counts with the observed variant and $n={n}_{1},{n}_{2},\dots ,{n}_{S}$ be the set of counts with the position covered, where $S$ is the number of cells. These statistics can be considered as a mix of variant counts arising from cells with actual mutations and cells with variant counts arising from technical sources (e.g., PCR and sequencing error). This can be modeled as a binomial mixture model $P\left(k|n,\Theta \right)={\uppi }_{1}B\left(k|n,{\uptheta }_{1}\right)+{\uppi }_{2}B\left(k|n,{\uptheta }_{2}\right)$, where ${\uptheta }_{1}$ is the probability of observing the variant in cells with the mutation, ${\uptheta }_{2}$ is the probability of observing the variant in cells without the mutation, ${\uppi }_{1}$ is the fraction of cells which have the mutation, and ${\uppi }_{2}$ is the fraction of cells which do not have the mutation. $B$ is the binomial probability distribution $\left(\genfrac{}{}{0pt}{}{n}{k}\right){p}^{x}{\left(1-p\right)}^{n-k}$. All the parameters of the binomial mixture model can be solved using an expectation maximization algorithm. The cells are split by XCI status, and the above binomial mixture model is applied to the two groups of cells separately. The mutation is considered to have passed the XCI status filtering if only one of the groups has a ${\uptheta }_{1}$ value above $0.04$. If both of neither have a ${\uptheta }_{1}$ value above $0.04$, the mutation does not pass the filtering. We also applied an absolute logit difference threshold of 1.22 to remove mutations where the ${\uptheta }_{1}$ value are close to each other but slightly above and below 0.04.

After filtering, the parameters can then be used to calculate the posterior probability of each cell harboring the mutation. In addition to calculating the posterior probability, we also perform a likelihood-ratio test. Briefly, the log-likelihood was calculated for ${\uptheta }_{1}$ and ${\uptheta }_{2}$, with the null hypothesis ${H}_{0}={\uptheta }_{1}$ and alternative hypothesis ${H}_{1}={\uptheta }_{2}$. In all cases, we assigned the null hypothesis to be ${H}_{0}=0.01$. The *p*-value is obtained by a chi-squared test with degree of freedom 1 and the test statistic $-2*\left(l{l}_{{H}_{0}}-l{l}_{{H}_{1}}\right).$ A cell is considered to harbor the mutation if the posterior probability is $>0.95$ and is considered significant in the likehood-ratio test at $\alpha = 0.05$. The relationship between sensitivity and specificity of the likelihood-ratio test was assessed using the Neyman-Pearson lemma [26].

### Assessment of mutation calling through simulations

To assess our ability to detect clonally informative mutation events across many scenarios, we simulated observations using the binom function from scipy stats. We simulated mutations at varying XCI ratio levels (0.1–0.9), heteroplasmy level (0.01–0.99), clonal fraction (0.001–0.1), and read coverage (10–500 reads) corresponding to realistic mutation discovery scenarios. The binomial mixture model was then applied to each XCI group separately. The sensitivity to detect a clonal mutations was found by calculating the logit difference between the maximum mutation probability found by the mixture model within each XCI group. A logit difference above 1.22 was considered a detection event, which is a cutoff used for analysis of real data.

### Mitochondrial mutation analysis of human PBMCs using mgatk

To obtain an initial set of mitochondrial mutations, *mgatk* (v0.6.6) was used to obtain the variant count and coverage matrices and to calculate metrics like variance mean ratio and strand correlation. The mutations were filtered by variance mean ratio > 0.01 and strand correlation > 0.65. The XCI-assisted mitochondrial mutation calling and filtering described above was applied.

For the PBMC donor 7, we found 766 mutations which passed basic filtering, 480 mutations which passed the XCI test, and 209 mutations which did not pass the XCI test but went through mutation calling as described above for both alleles. The remaining 77 mutations either failed because neither allele had a clonal heteroplasmy level above 4%, an absolute logit difference below 1.22, empirically determined to filter away artefactual observations, or the binomial mixture model failed to converge for both or one of the alleles.

### Mitochondrial mutation analysis of human PBMCs using Mquad

To obtain a set of mitochondrial mutations using *Mquad (v0.1.7)*,* cellSNP-lite (v1.2.3)* was first used to pile-up mtDNA variants from PBMC donor 7. We then applied *Mquad* and filtered the output variants using the *Mquad* quality filters PASS_KP and PASS_MINCELLS. We then applied the XCI assisted mitochondrial mutation calling and filtering as described above, which was then compared to the *mgatk* result.

### Analysis of colorectal *cancer* mtscATAC-seq data

The XCI status of each single cell was inferred as described above, and the mitochondrial mutations and UMAP co-ordinates were obtained from the supplementary repository of the article.

### Analysis of 10x GEX and VDJ data from a breast tumor

The scRNA-seq data from the breast tumor was obtained from the authors and the XCI status of each single cell was inferred as described above.

### Analysis of nuclear mutations using Monopogen

The software *Monopogen* (v1.6.0) was run for the PBMC donor 7 dataset. For the software to be compatible with smart-seq style data, we forked and modified the code at specific sections for data processing. We filtered the putative somatic mutations according to the instructions on github, SVM_pos_score > 0.5, LDrefine_merged_score > 0.25, Depth_alt > 1, and Depth_ref > 1. BAF_alt was filtered at < 0.5 and did not have a lower filter threshold. The single-cell genotype file produced by *Monopogen* was then used to classify mutations according to XCI status.

### Supplementary Information

Additional File 1: Supplementary figures.

Additional File 2: Review history

## Data Availability

The PBMC and mouse fibroblast sequencing data is available at the EBI European Nucleotide Archive (ENA) under accession numbers E-MTAB-7098 for the mouse fibroblast data and E-MTAB-11452 for the PBMC data [27, 28]. The mtscATAC-seq sequencing data from a colorectal tumor sample was downloaded from GEO with accession number GSE142745 [29], and the mitochondrial mutations and UMAP coordinates were obtained from the supplementary repository of the original article [30]. The immune receptor sequences and UMAP coordinates for the breast tumor data were obtained from the supplementary repository of the manuscript [31]. Processed files generated by this study may be found on Zenodo [32]. Code for data analysis and reproducing the plots are deposited on Zenodo and available on GitHub under the MIT license [33, 34]. The forked version of Monopogen is also available on Zenodo and GitHub under the GPL-3.0 license [35, 36].
