# Supplementary material for: Inferring clonal somatic mutations directed by X chromosome inactivation status in single cells
Source: Genome Biol. 2024 Aug 9;25:214. doi: 10.1186/s13059-024-03360-1 (PMC11312698; doi:10.1186/s13059-024-03360-1)
Supplement: Supplementary file 1 — Additional File 1: Supplementary figures. [file 13059_2024_3360_MOESM1_ESM.docx]

**Additional File 1: Supplementary Figures**

**
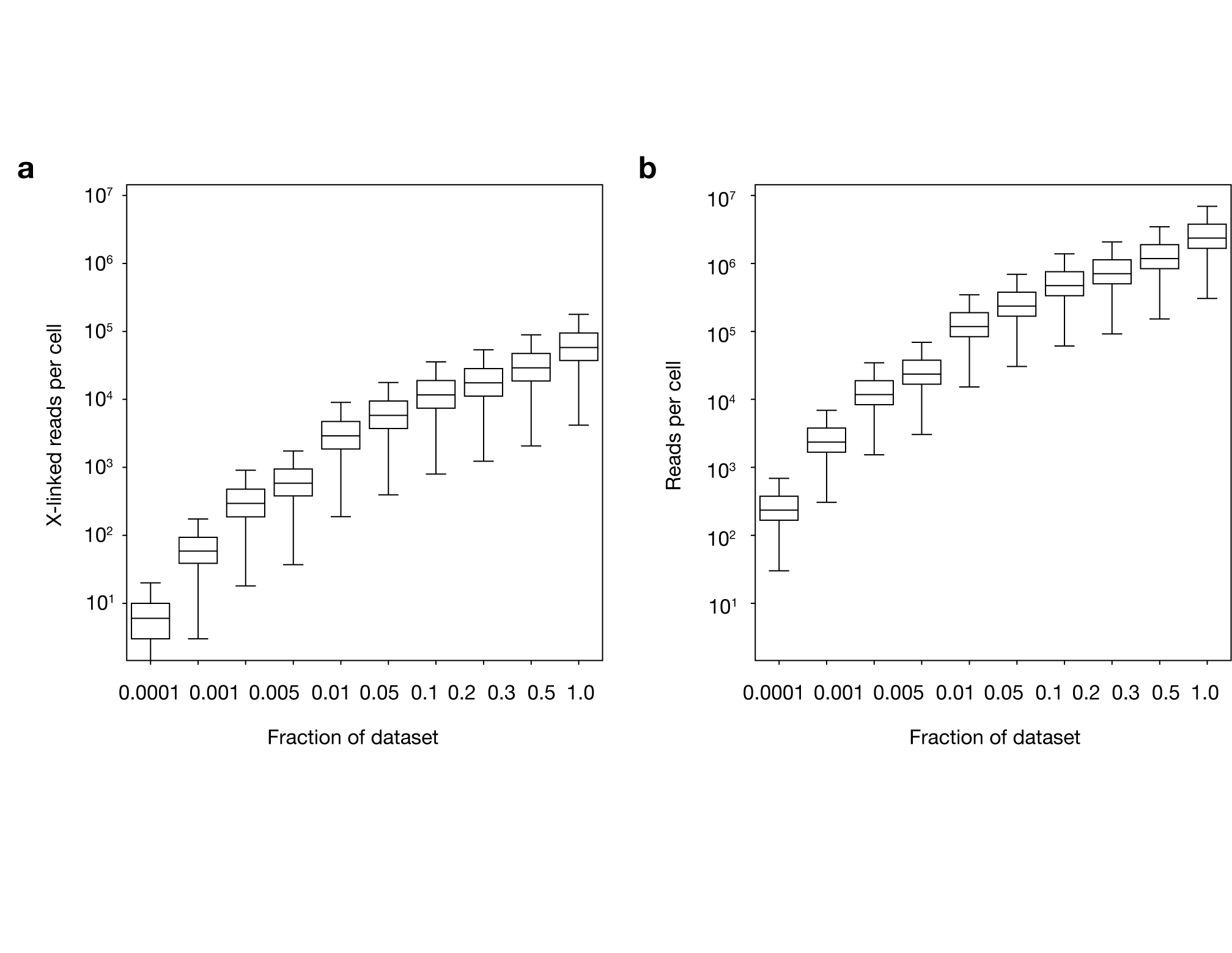
**

**Fig S1. Downsampling of reads required for detection of X-linked variants.** **a**, Number of X-linked reads per cell for each downsampled condition. **b**, Total number of reads per cell for each downsampled condition, n = 232 cells. Centre lines denote the median; hinges denote the first and third quartiles; whiskers denote 1.5× the interquartile range (IQR).


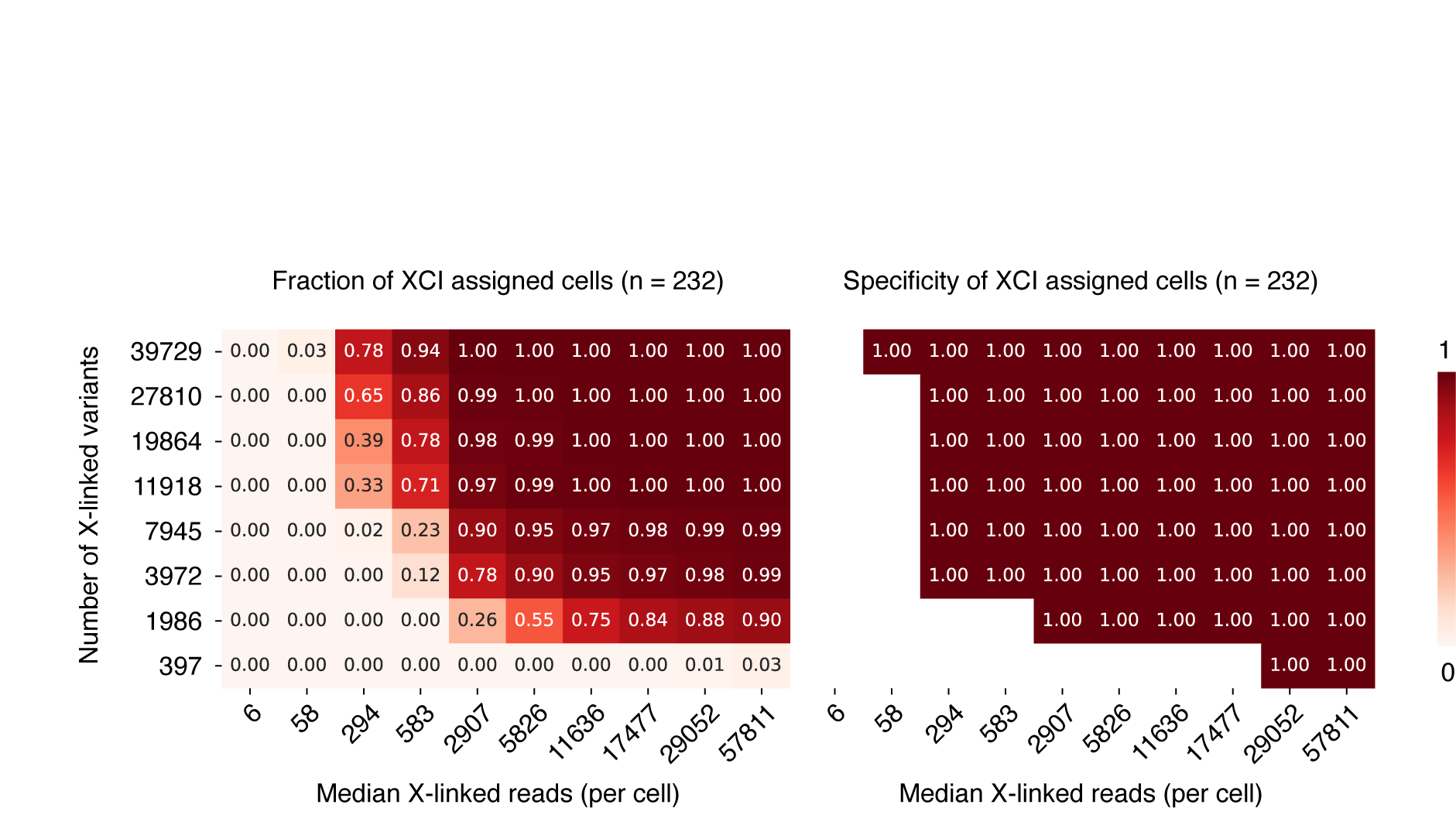


**Fig S2. Sensitivity and specificity of XCI assignment strategy using vireo.** **Left**: Heatmap showing the fraction of cells which was assigned an XCI status, as a function of the median number of X-linked reads (x-axis) and number of X-linked variants used by vireo (y-axis). **Right**: Heatmap showing the fraction of cells which was were correctly assigned XCI status out of the cells which was assigned a status, as a function of the median number of X-linked reads (x-axis) and number of X-linked variants used by vireo (y-axis).


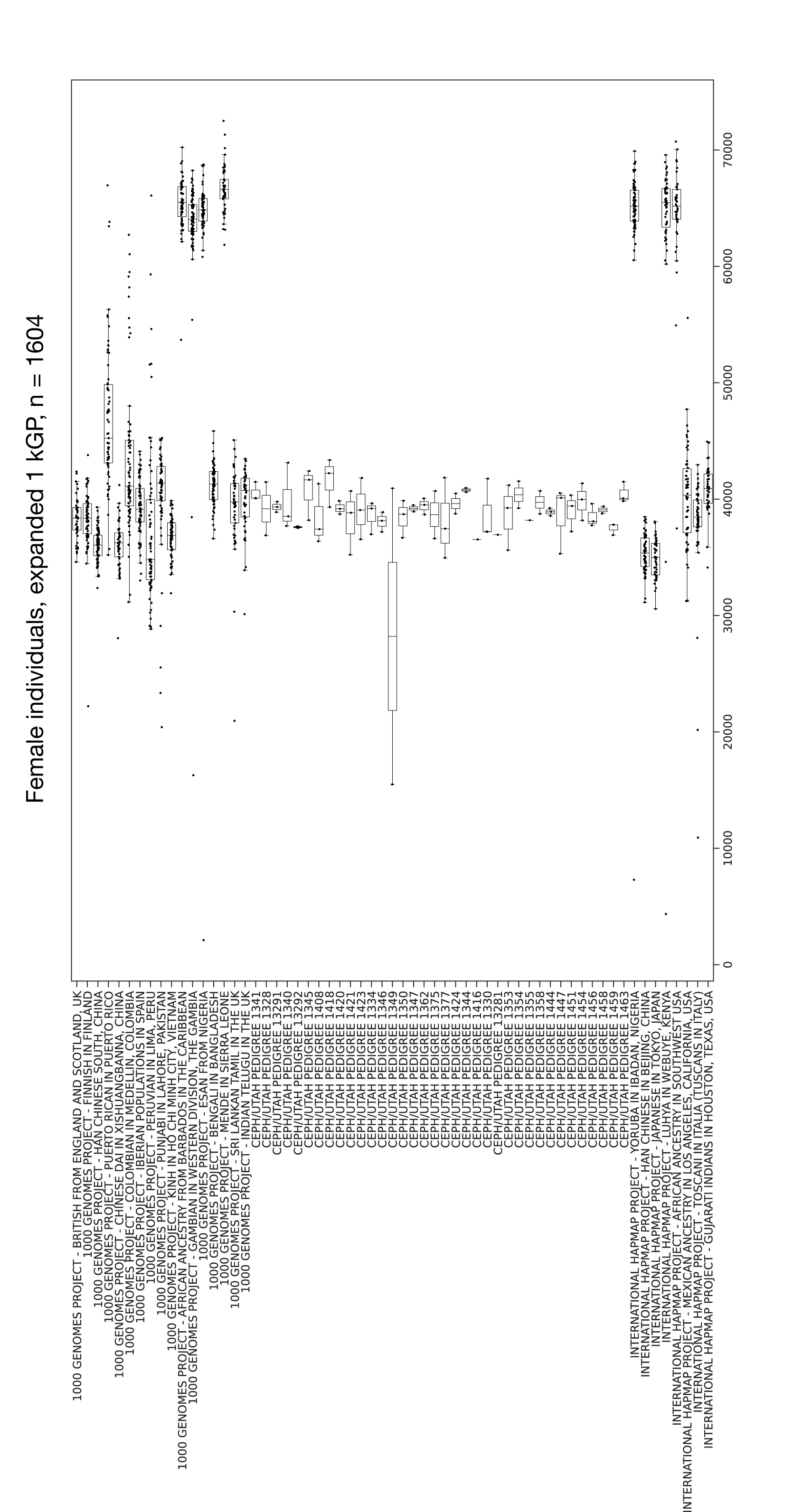


**Fig S3. X-linked heterozygous SNPs in genic regions.** Number of X-linked heterozygous SNPs in genic regions for each population or trio in the expanded 1kGP dataset. Total of 1,604 individuals.

**
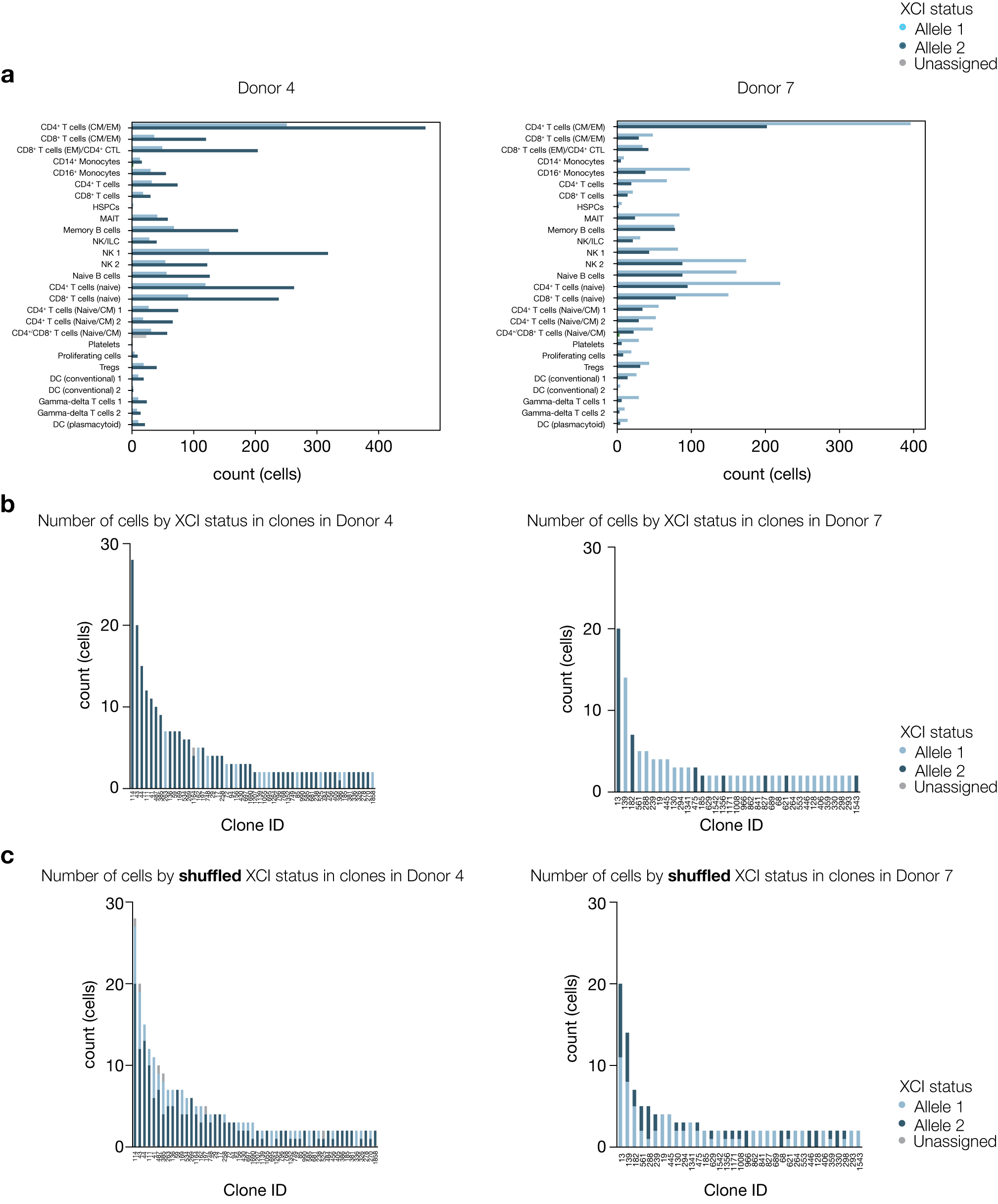
**

**Fig S4. Distribution of active X alleles in distinct cell types and clones.** **a**, Number of cells with a predicted XCI status for each cell type cluster in Donor 4 (left) and Donor 7 (right). **b**, Stacked bar plot of XCI status of cells in T-cell clones as defined by their TCR sequences for Donor 4 (left) and Donor 7 (right). **c**, Stacked bar plot of shuffled XCI status of cells in T-cell clones as defined by their TCR sequences for Donor 4 (left) and Donor 7 (right).


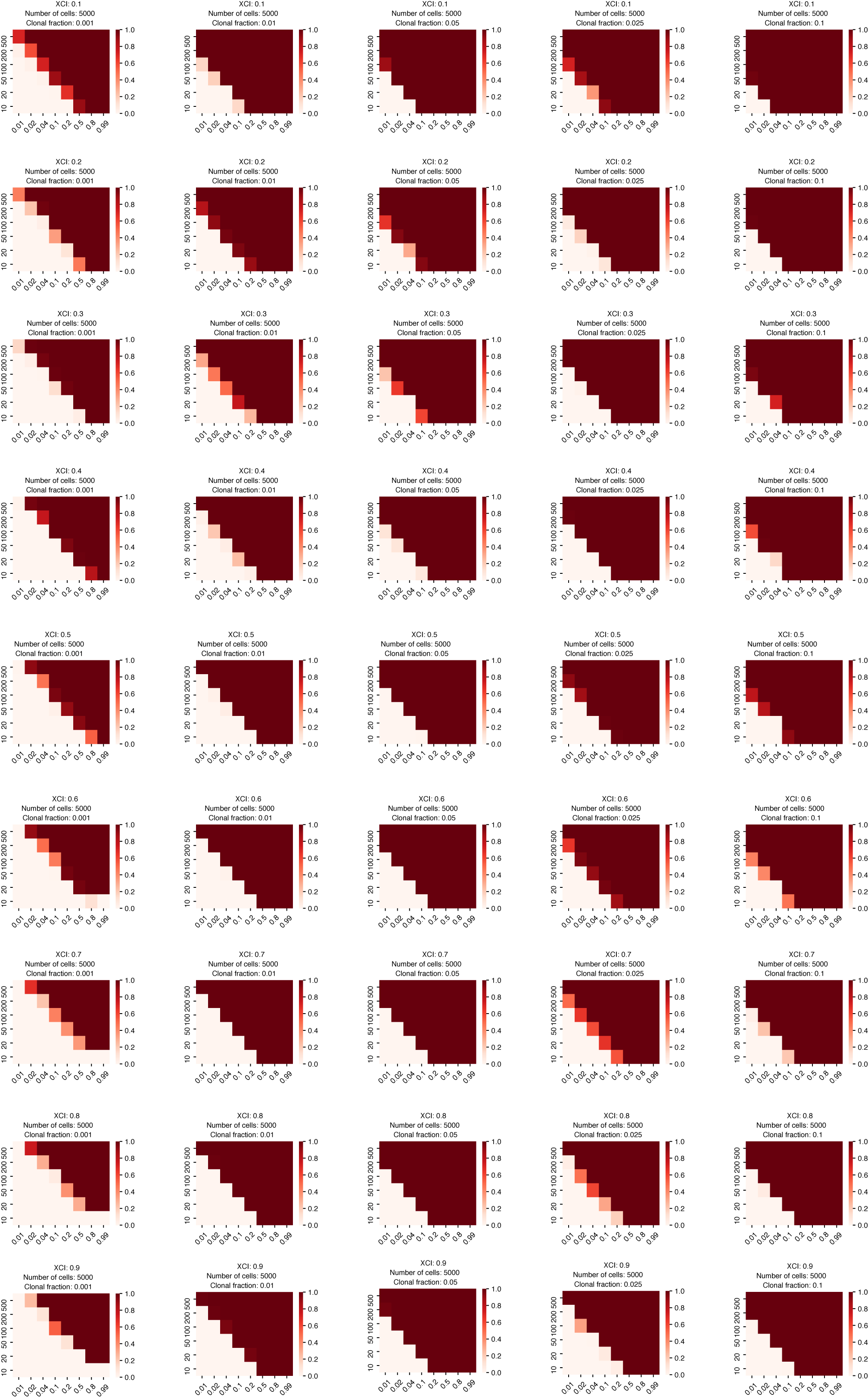


**Fig S5. Simulations to assess power to detect clonal mutations.** Each heatmap asseses the ability to detect clonal mutations given a specific set of relevant factors. Heatmaps in each row have the same XCI ratio for the tested group, and heatmaps in each column have the same clonal fraction. The specific values are present in the top of each individual heatmap. The x-axis of each heatmap show the tested heteroplasmy level of the cells harboring the mutation, while the y-axis show the number of reads covering the mutation site. The color is the fraction of simulations the mutation was detected in the population.


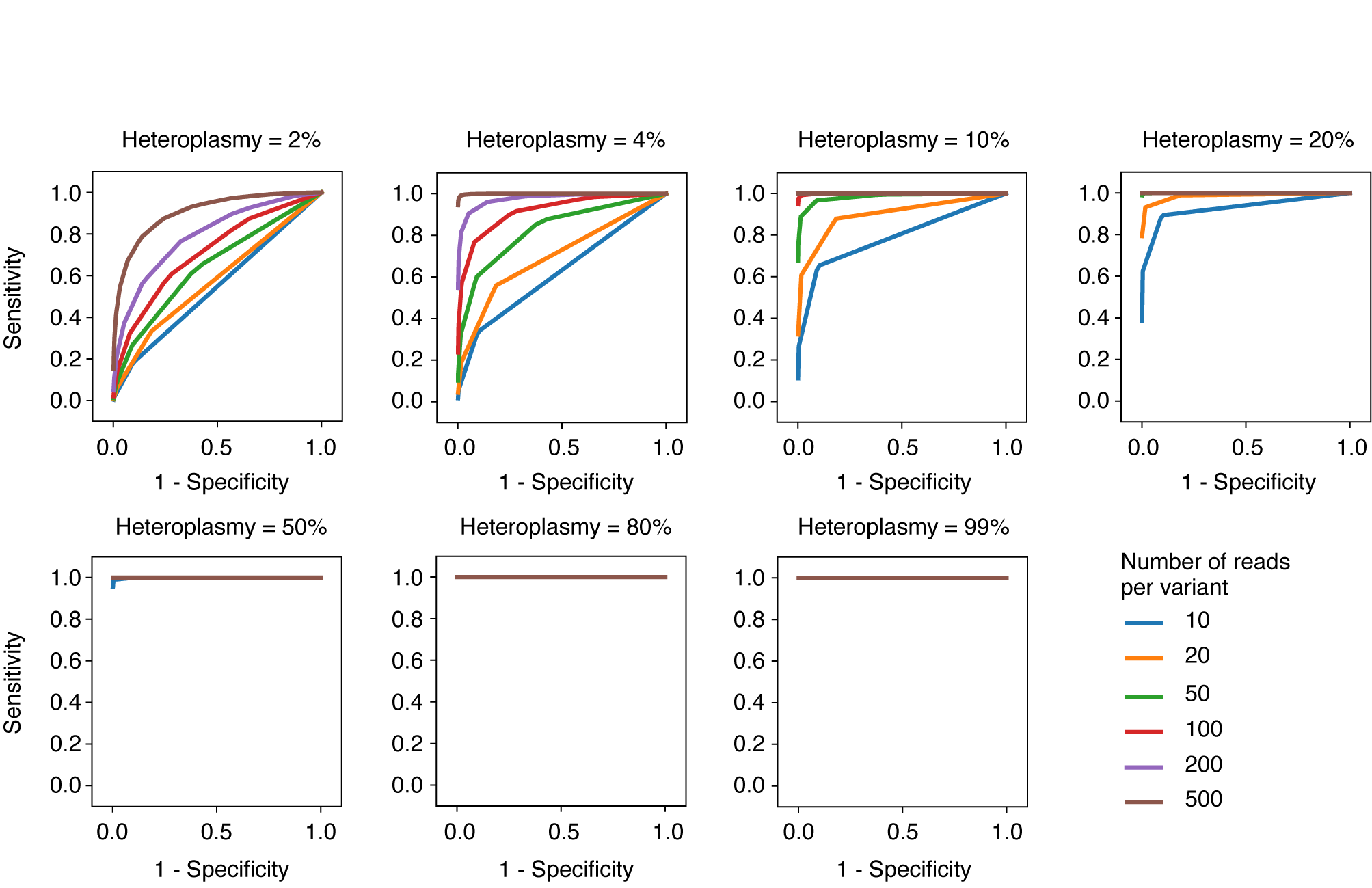


**Fig S6. Sensitivity and Specificity of likelihood-ratio test for mutation calling using the binomial mixture model.** Specificity plotted as a function of sensitivity for different heteroplasmy level (2%, 4%, 10%, 20%, 50%, 80%, 99%) and different number of reads covering the variant position (10, 20, 50, 100, 200, 500).


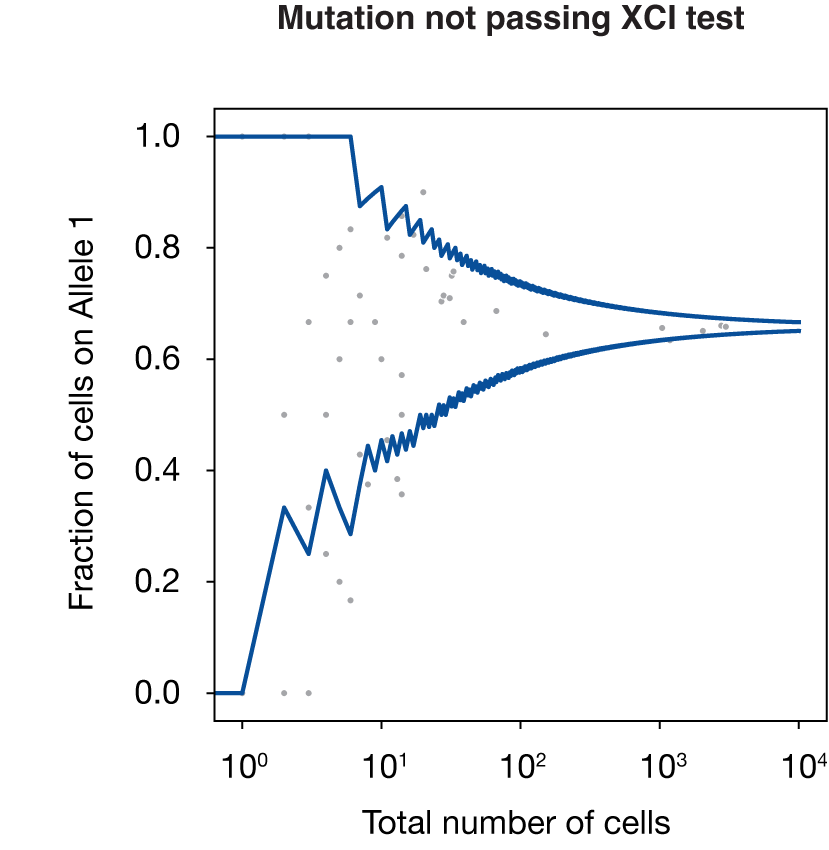


**Fig S7. Confirmation of non-clonal mitochondrial mutations.** Fraction of cells with Allele 1 as the active X chromosome compared to the total number of cells with the detected mutation, for mitochondrial mutations which did not pass the XCI based filtering (see Fig 2h). The blue lines are 5th and 95th percentile of randomly sampling cells with Allele 1 active based on the binomial distribution and p = 0.66 (the fraction of cells with Allele 1 active in the dataset), n = 209 mutations.

**
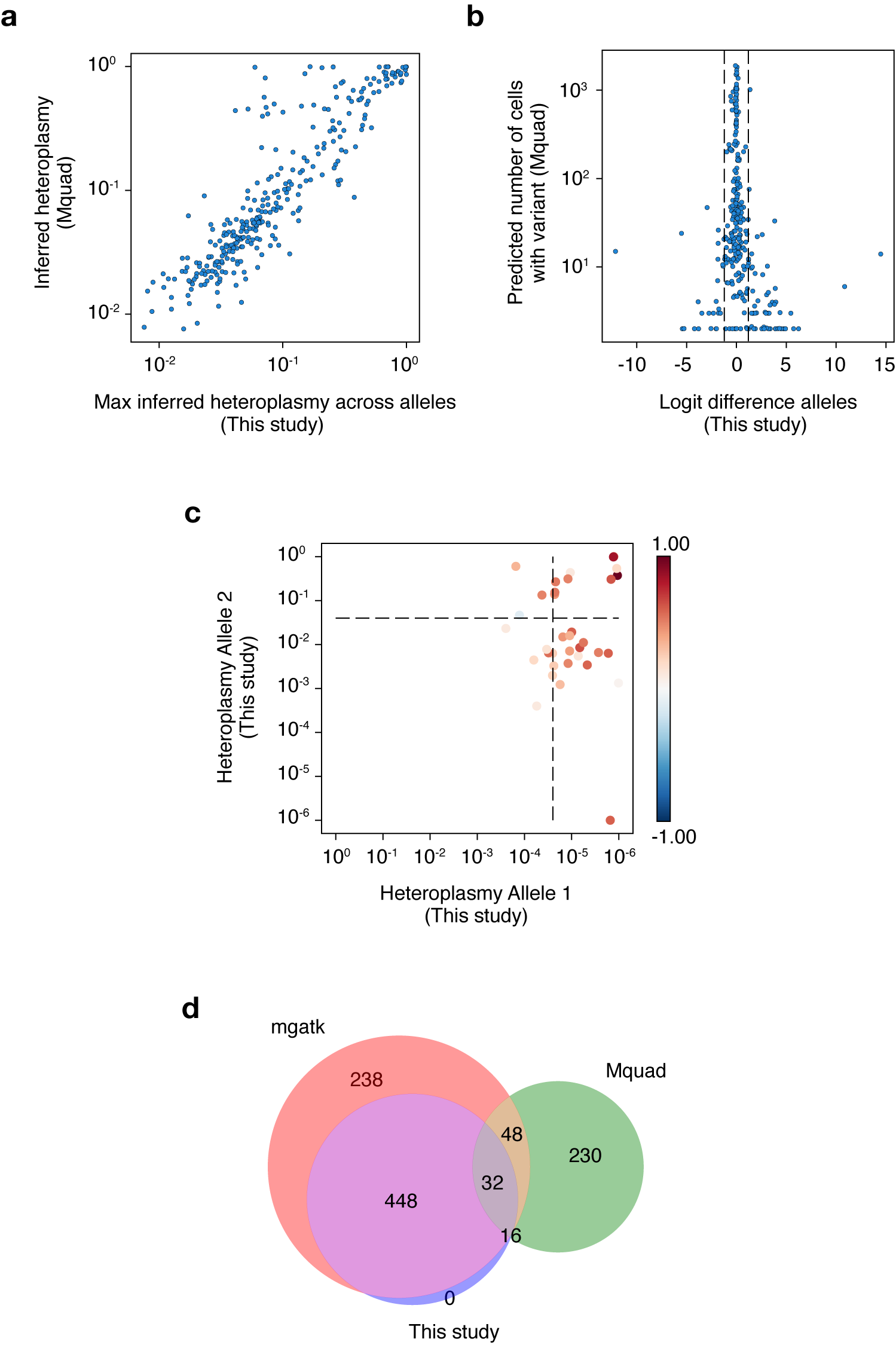
**

**Fig S8. Identifying clonal mutations using Mquad. a**, The maxmimum heteroplasmy level detected across XCI groups compared to heteroplasmy detected by Mquad (n = 310 variants). **b**, The logit difference between XCI groups compared to the number of cells with the mutation as predicted by Mquad. The dashed lines are at -1.22 and 1.22 respectively, n = 310 variants. **c**. The clonal heteroplasmy levels of mutations only detected by Mquad, colored by the strand correlation metric as measured by mgatk, n = 35 variants. **d**. Venn diagram showing detected variants by mgatk, Mquad and this study.

**
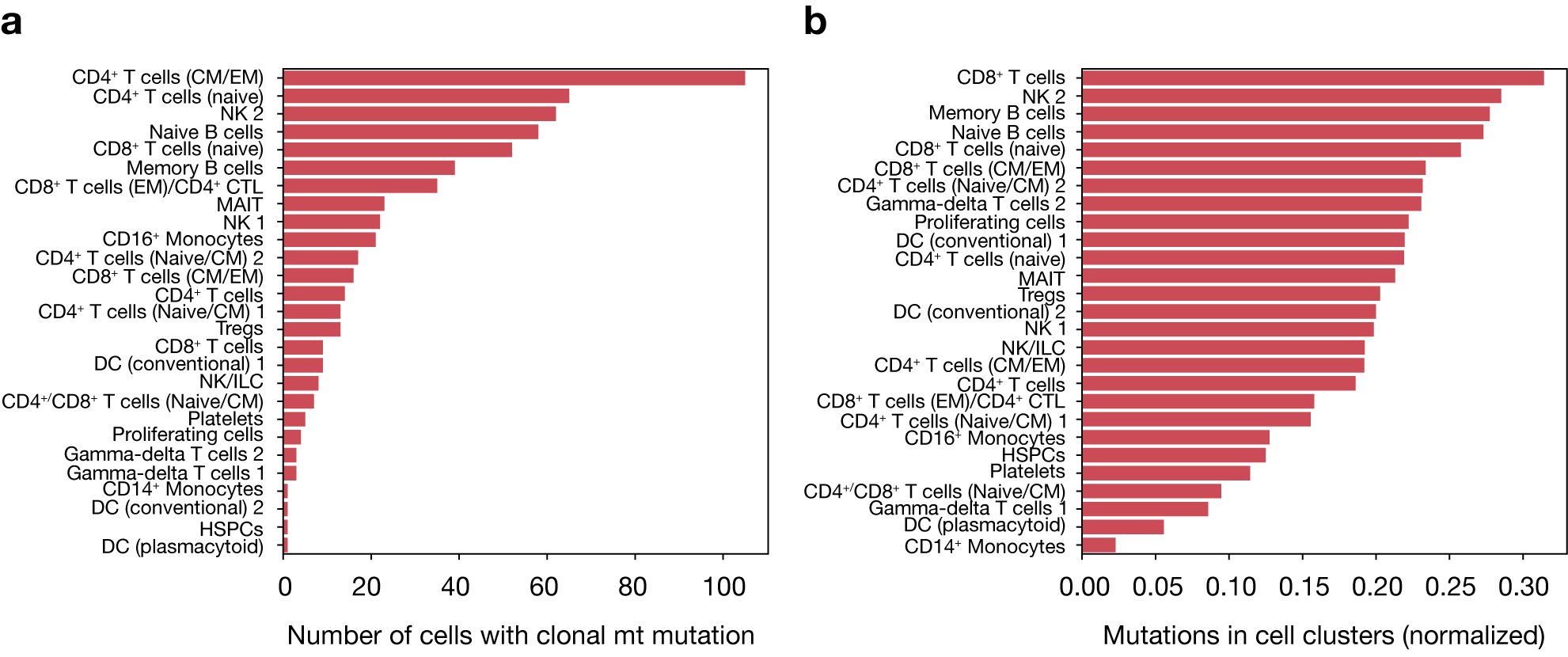
**

**Fig S9. Count distribution of mitochondrial mutations per cell type in Donor 7. a**, Number of cells with clonal mutations per cell type cluster, sorted from highest to lowest. **b**, Number of mutations in each cell cluster normalized by the number of cells in each cluster, sorted from highest to lowest.

**
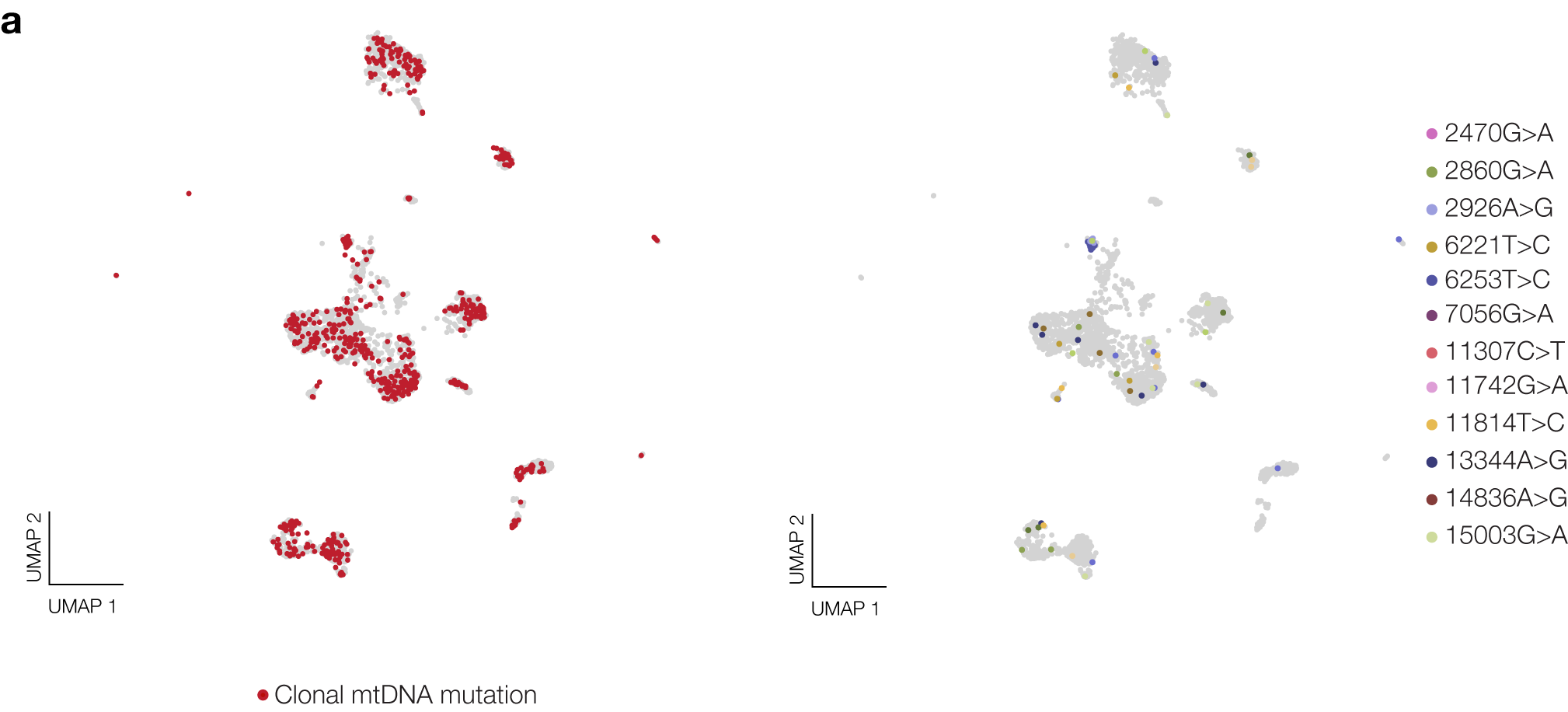
**

**Fig S10. Distribution of clonal mitochondrial mutations across cell types.** Cells with clonal mitochondrial mutations visualized on the UMAP space (**left**) and the 12 most abundant clonal mutations visualized (**right**).

**
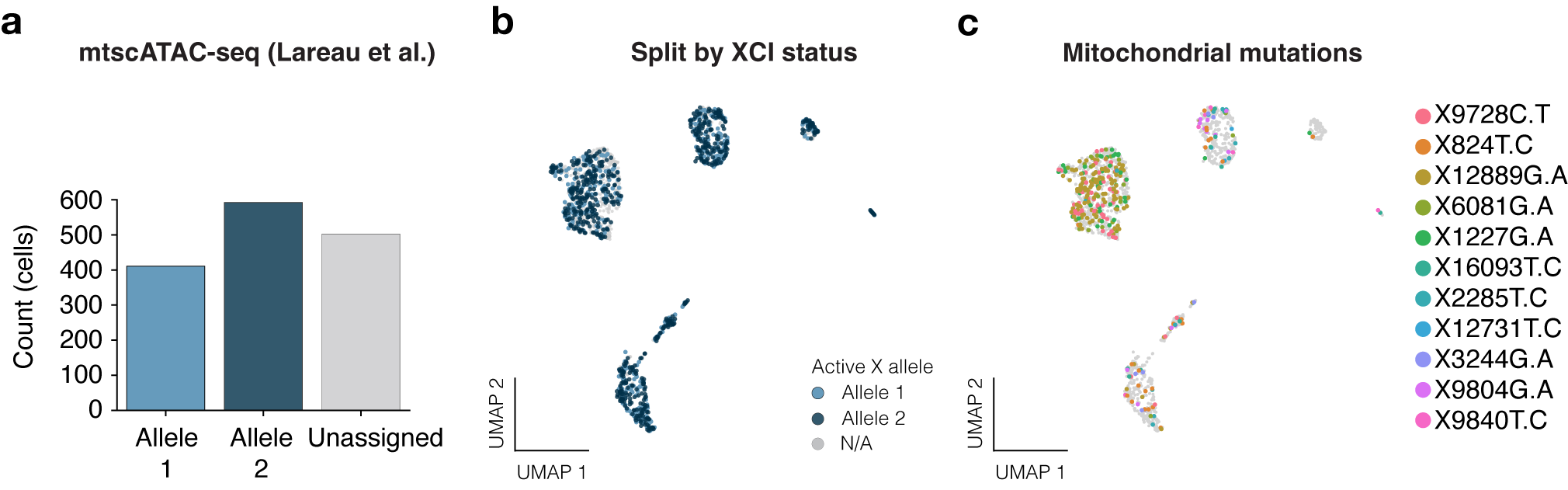
**

**Fig S11. Application of X chromosome inactivation inference to single-cell ATAC-seq enriched for mitochondrial mutations. a**, Deconvolution of active X-alleles and assignment of XCI status to single cells in colorectal cancer. **b**, Visualization of XCI status in single cells with UMAP. **c**, All mitochondrial mutations passing 5% heteroplasmy threshold visualized with UMAP in single cells.

**
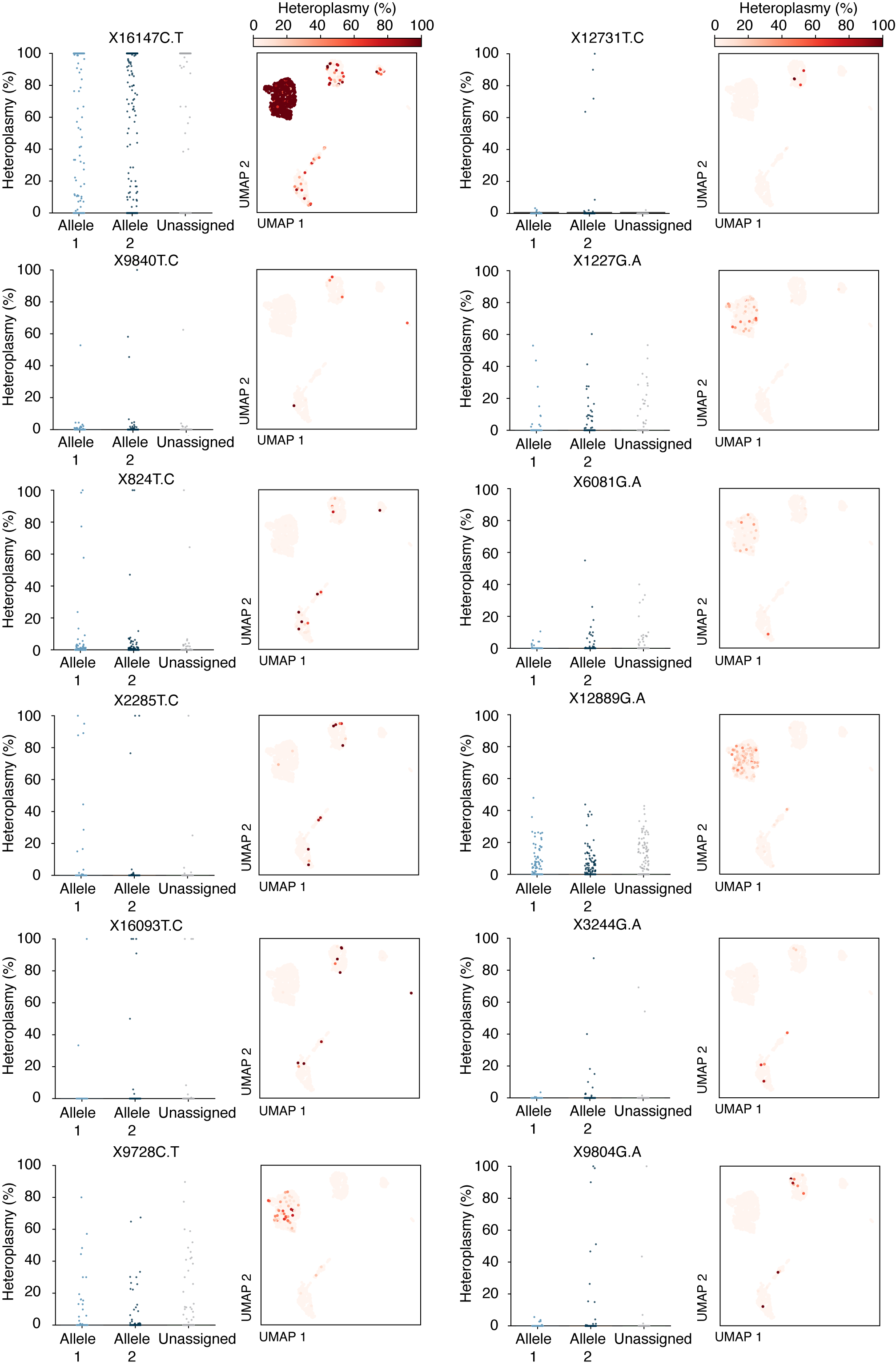
**

**Fig S12. X chromosome inactivation status of cells with mitochondrial mutations detected in single cells.** Heteroplasmy levels of mutations in single cells separated by active X-allele and visualized with UMAP.


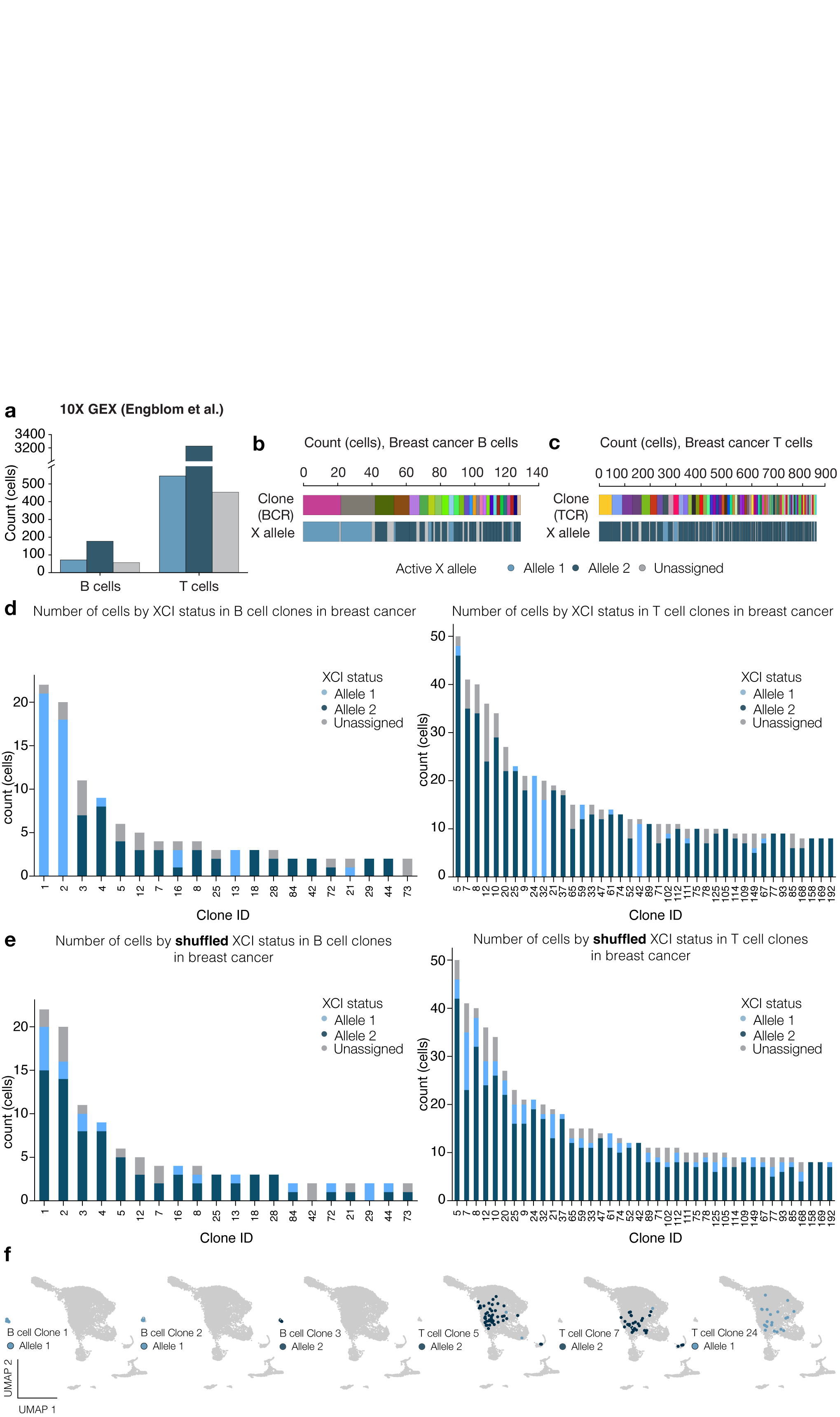


**Fig S13. Application of X chromosome inactivation inference to 10X single-cell RNA-seq. a**, Deconvolution of active X-alleles and assignment of XCI status to single cells in breast cancer. **b**, Clonality of cells based on B cell receptor sequences compared with their XCI status. Colors denote individual B cell clones. **c**, Clonality of cells based on T cell receptor sequences compared with their XCI status. Colors denote individual T cell clones. **d**, Stacked bar plots of XCI status for the 20 largest B cell clones (left) and 40 largest T cell clones (right). **e**, Stacked bar plots of shuffled XCI status for the 20 largest B cell clones (left) and 40 largest T cell clones (right). **f**, Visualization of the largest 3 clones in B and T cells with UMAP. Cells are colored by their XCI status.

**
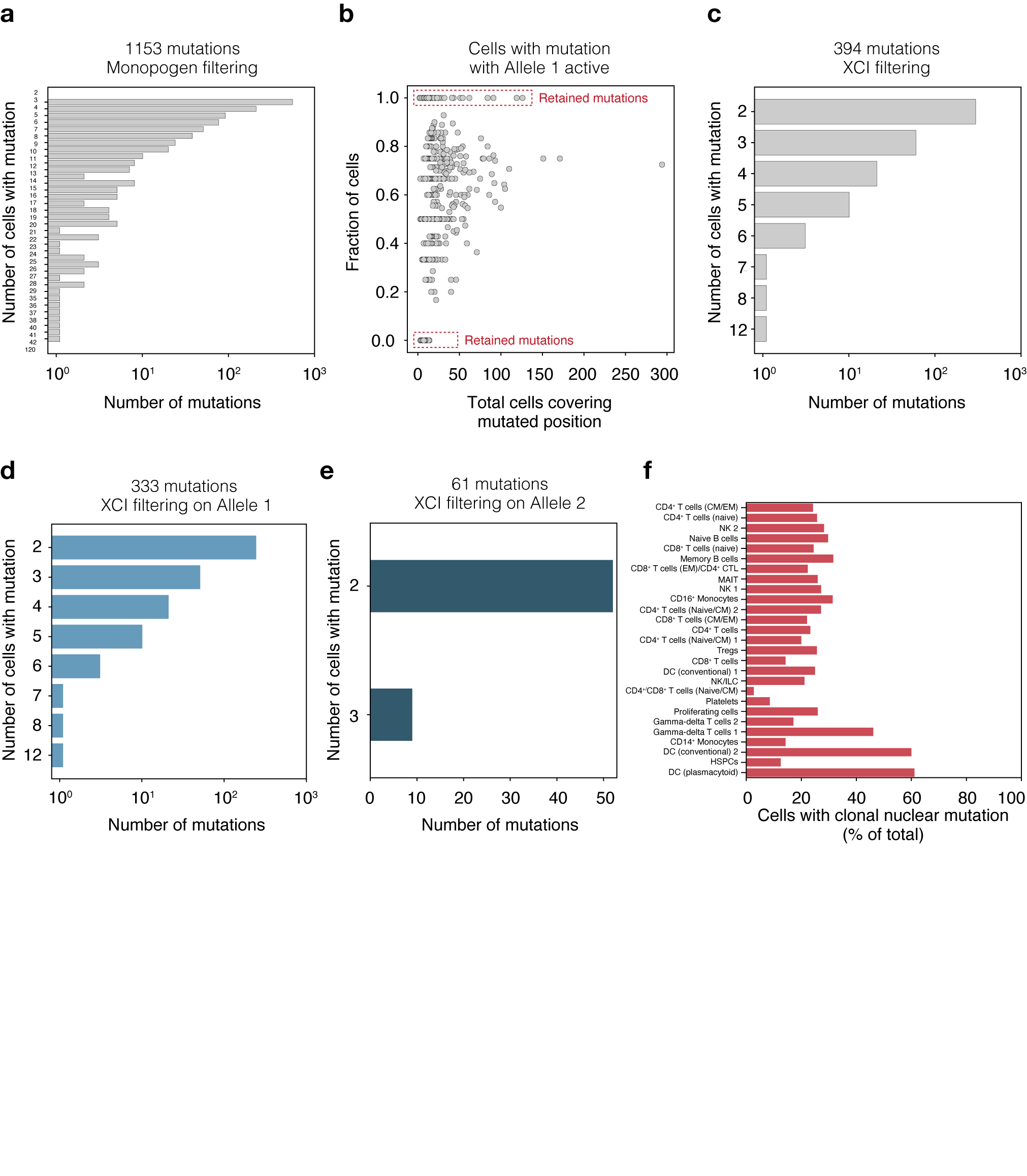
**

**Fig S14. Discovery of clonally informative nuclear SNVs using Monopogen and XCI directed filtering. a**, The number of mutations (x-axis) which are detected in a given number of cells (y-axis), n = 1,153 mutations, Donor 7. **b**, The fraction of cells with allele 1 active (y-axis) and the total number of cells with coverage over the mutated position (x-axis). The mutations in the red dashed boxes are retained for further analysis, n = 1,153 mutations total, 333 mutations in top red box and 61 mutations in bottom red box. **c**, The number of mutations (x-axis) which are detected in a given number of cells (y-axis) after XCI directed filtering, n = 394 mutations. **d**, The number of mutations (x-axis) which are detected in a given number of cells (y-axis) after XCI directed filtering for allele 1, n = 333 mutations. **e**, The number of mutations (x-axis) which are detected in a given number of cells (y-axis) after XCI directed filtering for allele 2, n = 61 mutations. **f**, Percentage of PBMCs with nuclear mitochondrial mutations for each cell type from Donor 7.
