# Supplementary material for: Inferring clonal somatic mutations directed by X chromosome inactivation status in single cells
Source: Genome Biol. 2024 Aug 9;25:214. doi: 10.1186/s13059-024-03360-1 (PMC11312698; doi:10.1186/s13059-024-03360-1)
Supplement: Supplementary file 2 — Additional File 2: Review history [file 13059_2024_3360_MOESM2_ESM.docx]

**1^st^ round**

**Reviewer 1**

The manuscript presents a novel method utilizing X chromosome inactivation (XCI) status to identify informative mutations, thereby assisting in the delineation of clonal cell structures. Initially, the author employs a Bayesian XCI deconvolution method to discern paternal and maternal alleles and determine XCI status for each cell. In the next step, the author developed a statistical method that leverages XCI status in each cell to identify informative mutations. This statistical method involves categorizing cells by their XCI status and applying a binomial mixture model to detect mutations and assign a probability of mutation presence in individual cells. This approach not only identifies mutations but also assigns posterior probabilities of mutation presence in single cells, representing an advancement over previous methods that used similar models. Overall, it is a good idea with largely valid results.

Major Concerns.

1. Systematic evaluation of the algorithm's performance is lacking:
The manuscript currently lacks systematic evaluations of the algorithm's performance.
- It is essential to conduct a detailed evaluation analysis of algorithm's specificity and sensitivity across various conditions.
- An in-depth discussion of the trade-offs between precision and recall is needed.
- The systematic evaluation should be performed on both Bayesian XCI deconvolution and mutation filtering steps.
-
2. Comparative analysis with established methods is lacking:
The manuscript should include benchmarks against existing methods such as, MQual (Nature Communications), maegatk (mitochondrial genome variant calling applied by Maester paper), highlighting its potential advantages or limitations. Ideally, they should show it using existing real datasets, or alternatively, they should elucidate their advantage through simulations.

3. Parameters and biological background
What are the expected ratios of allele 1 and allele 2 sites in different cell types/donors? It seems that the ratio can have a wide range (Sup. Fig. 9a-c) in different cell types. When the ratio is near 0 or 1, it may become difficult to delineate the paternal vs the maternal cells. Have the investigators examined this systematically and had any advice in what biological context the approach is sufficiently powerful to be applied?

4. Improve writing and clarity:
The writing is a bit sketchy and there is a bit of disconnect from text to figures. For example,
- The manuscript states (Line 59 - 61), "Further visualization of the most abundant T cell clones and their respective XCI status showed that effector memory CD8+ T cell population shows the highest level of clonal expansion in both donors, and mucosal associated T cells in Donor 7 (Fig. 2d)." However, Figure 2d does not provide explicit labeling of cell types, making it challenging to verify the author's conclusions regarding clonal expansion in specific T cell populations. Clarification or additional labeling in Figure 2d is necessary for readers to accurately assess these findings.
- The manuscript states (Line 103- 105), "Finally, we found that gamma-delta T cells do not share mutations with other T cell lineages, suggesting these subsets are produced by a separate set of progenitors than CD4+ and CD8+ T cells." However, similar observations of low relative covariance between these cell types are also evident in fate coupling analysis using other filtering criteria, as shown in Figures 2j and 2k. This indicates that the conclusion regarding gamma-delta T cells is not exclusive to the method presented in this study. It would be beneficial for the authors to acknowledge this and discuss how their method's findings align with or differ from these existing observations. Such a comparison would provide a clearer understanding of the novel contributions and potential limitations of the proposed method.
- Conclusions of Figure 2i (Line 98 to Line 103) about the lineage relationships and mutation sharing among T cell subsets are not directly interpretable from the figure. For example, the statement 'naïve CD4+ T cells were not coupled with a subset of CD4+ CM/EM T cells' (Line 100) appears to be contradicted by the high covariance observed between these two cell types in Figure 2i. This discrepancy between the text and the figure raises questions about the accuracy of the conclusions drawn.
- The manuscript does not sufficiently contextualize these findings within the broader scope of existing research. A discussion comparing these observations with previous studies or established knowledge in the field is necessary. This would help in understanding how these new findings align with or challenge the current understanding of T cell lineage differentiation and development.
- Finally, the discussion should extend beyond the immediate results to explore the broader implications of these findings. How do they contribute to our understanding of T cell development and differentiation? Are there potential clinical or research applications that these findings could inform?

5. Improve Application Scope:
Can the approach be generalized to broader settings?
- The method's reliance on X chromosome inactivation (XCI) inherently limits its use to female samples. However, can the knowledge (e.g., mutation sites) learnt be generalized to male samples?
- Can the approach be generalized to genomic RNA?

Minor Concerns:
1. Mislabeling of Figure Legends in Fig 2h and g:
The legend descriptions for parts 'h' and 'g' appear to be swapped in relation to their corresponding plots. (Line 319 - 321)

2. Clarification Needed for Figure 2d Labeling:
In the manuscript, there is a reference to Figure 2d for the visualization of the most abundant T cell clones and their XCI status, specifically mentioning effector memory CD8+ T cells and mucosal-associated T cells in Donor 7 (Line 59 - 61). However, I noticed that the cell types are not explicitly labeled in Figure 2d. Please add clear labels to Figure 2d to accurately reflect the cell types being discussed.

3. Concerns on threshold selection in the binomial mixture model:
In the manuscript, the threshold for mutation filtering in the binomial mixture model is set at 0.04 (Line 219), but the rationale behind this specific value is not clear. It would be beneficial for the author to provide justification for this choice, particularly considering its impact on the accuracy and reliability of the model in different datasets and biological contexts. An optimally set threshold is crucial to minimize the risk of false positives or negatives. Therefore, a more detailed justification or a method for determining this threshold in different research scenarios would be highly beneficial.

4. Concerns Regarding the Claim of an "Unbiased" strategy:
I would like to address the use of the term "unbiased" as mentioned in the Discussion section (line 138). In statistical inference, "unbiased" typically refers to methods or estimators that are demonstrably free from systematic error, meaning their expected value is equal to the parameter being estimated.
- For a claim of unbiasedness to be substantiated, it is generally expected that there be either a formal mathematical proof or a comprehensive set of simulation studies. These should demonstrate the estimator's unbiased nature across a variety of scenarios and parameter settings.

- As an alternative or in addition, a comparative analysis with other established methods, highlighting the relative absence of bias in the proposed method, would be valuable. This would provide a clearer understanding of the method's position in terms of bias compared to existing approaches.

**Reviewer 2**

In this work, authors show that by leveraging the information about the X chromosome inactivation, they get rid of some of the noise typical in somatic mutation calling and improve the analysis of mitochondrial mutations in single cells coming from females. The idea of identifying the X chromosome inactivation status is simple, but very useful. For example, to later identify the clonal evolution, the input signal from just one chromosome is cleaner.

Major comments
The method the Authors use is not theirs. They use vireo
https://genomebiology.biomedcentral.com/articles/10.1186/s13059-019-1865-2
Vireo is a powerful approach for identifying genotypes in single cell data and can be used for various applications, such as demultiplexing. However, this approach is not new, the method has been previously published and is widely used.

On top of vireo, the authors use a binomial mixture model to call activated chromosome mutations. It is a very simplistic model. In particular, it accounts for the false positive error rate - the prob. that the mutation which is not present in reality, will be observed due to technical noise. In fact, the opposite may also happen (existing mutation will not be observed). In most generality, there are four different nucleotides that can be observed and only one of them is true. The authors do not consider that at all and should at least mention this as a limitation.
On the side of the methodology, it is really nothing impressive.

Minor
The authors in their procedure use an arbitray cutoff of 0.04 for \theta_1 and \theta_2. Please add more clarification to motivate the use of this particular cutoff.

**Authors’ response**

Reviewer 1

The manuscript presents a novel method utilizing X chromosome inactivation (XCI) status to identify informative mutations, thereby assisting in the delineation of clonal cell structures. Initially, the author employs a Bayesian XCI deconvolution method to discern paternal and maternal alleles and determine XCI status for each cell. In the next step, the author developed a statistical method that leverages XCI status in each cell to identify informative mutations. This statistical method involves categorizing cells by their XCI status and applying a binomial mixture model to detect mutations and assign a probability of mutation presence in individual cells. This approach not only identifies mutations but also assigns posterior probabilities of mutation presence in single cells, representing an advancement over previous methods that used similar models. Overall, it is a good idea with largely valid results.

*We thank the reviewer for the positive comments and the interest in our work.*

R1 Major Concern 1:

Systematic evaluation of the algorithm's performance is lacking: The manuscript currently lacks systematic evaluations of the algorithm's performance.

- It is essential to conduct a detailed evaluation analysis of algorithm's specificity and sensitivity across various conditions.

- An in-depth discussion of the trade-offs between precision and recall is needed.

- The systematic evaluation should be performed on both Bayesian XCI deconvolution and mutation filtering steps.

*Thank you for this important point. In the revised manuscript, we have shown the sensitivity of the Bayesian XCI deconvolution step in a new figure. New text in the revised manuscript:*

*The approach maintains the high recovery of XCI status for all high-quality cells down to 3,000 X-linked reads (120,000 reads total,* ***Supplementary Figure 1b****) and 12,000 heterozygous genic SNPs, after which the performance becomes worse (****Fig. 1d****). Moreover, we found that in all cases the XCI status was either correctly assigned or left unassigned (****Supplementary Figure 2****).*

*Furthermore, in a new figure we have assessed the sensitivity of the mutation refinement step based on XCI status across multiple relevant parameters using simulations, included below. Moreover, we have included an evaluation of mutation calling sensitivity and specificity based on the binomial mixture model we have used. New text in the revised manuscript:*

*We assessed our approach to detect clonal mutations using a simulation approach which modelled many possible factors that might impact sensitivity of detection. The factors included XCI ratio, number of cells profiled, the fraction of cells in the population with the mutation, heteroplasmy level of the mutation, and the number of sequencing reads covering the variant. The main factors that impacted sensitivity were heteroplasmy level and read depth. XCI ratio did not have an impact on sensitivity. At the read depths commonly used for mitochondrial mutation discovery (100x coverage), our approach is generally able to detect clonal mutations at a heteroplasmy level of 4% and above (****Supplementary Figure 5****). Moreover, we also assessed the sensitivity and specificity of mutation calling in single cells using the binomial mixture model and we found excellent sensitivity at high specificity at heteroplasmy level 4% and above (****Supplementary Figure 6****).*

*The revised manuscript also includes a discussion of the trade-offs between precision and recall in the context of analyzing clonal relationships between cells:*

*Here we demonstrated that using XCI status is an effective strategy to select clonally informative mutations for analyses, that effectively removes more widespread mutations or artefactual observations. The use of somatic variation to assign clonal relationships have a clear trade-off between precision and recall. Current methods to detect and assign somatic mutation events as marking related cells have limited ways to confirm that the discovered mutations occurred during an appropriate timeframe for clonal analysis. The approach introduced here significantly increases the precision in the sense that the selected mutations are guaranteed to have been acquired at a later point in development. One clear limitation of this approach is the inability to identify of sub-clonal relationships between cells which have undergone a bottleneck event after XCI. For example, identifying sub-clonal mutations in B or T cells will not be helped by the XCI approach, since these cells all already share XCI status. Another example would be identifying clonal mutations within cancers which originate from a single cell.*

R1 Major Concern 2:

Comparative analysis with established methods is lacking:

The manuscript should include benchmarks against existing methods such as, MQual (Nature Communications), maegatk (mitochondrial genome variant calling applied by Maester paper), highlighting its potential advantages or limitations. Ideally, they should show it using existing real datasets, or alternatively, they should elucidate their advantage through simulations.

*Mquad and maegatk/mgatk perform preprocessing and mutation selection but does not assign mutation probabilities to individual cells. The purpose of our approach is to perform further refinement of the mutations selected by tools such as Mquad or mgatk. In the initial manuscript, we used the filtering guidelines suggested by mgatk to select variants we then examined for XCI status.*

*In response to this question, we have extensively extended the comparison to existing methods. We now perform an explicit comparison to both mgatk and Mquad with regards to the mutation refinement based on XCI status and their agreement in a real dataset (PBMCs). New text in the revised manuscript:*

*We also compared this set of mutations to the mutations obtained by Mquad. Mquad identified 310 variants using an approach orthogonal to mgatk based on model-based identification using the binomial mixture model. Reassuringly, the heteroplasmy levels of mutations found by Mquad highly agreed with the maximum heteroplasmy level across alleles found by our approach (****Supplementary Figure 8a****). By computing the logit difference between the inferred heteroplasmy of the two alleles, we found that many of the variants found by Mquad had similar heteroplasmy levels on both regardless of XCI status, and 82 variants remained after this filtering based on this metric. Interestingly, this procedure removed all the variants found in many cells in the population (****Supplementary Figure 8b****). Out of the 82 mutations, 35 were only found by Mquad, and 16 of these mutations passed the same criteria we established for mgatk. These variants generally had high strand correlation which was slightly below the 0.65 cut-off, one of the quality criteria used by mgatk to identify variants (****Supplementary Figure 8c****). Overall, the Mquad and mgatk discovered a surprising number of different variants, although most variants discovered only by Mquad did not pass our filtering (****Supplementary Figure 8d****).*

R1 Major Concern 3:

Parameters and biological background:

What are the expected ratios of allele 1 and allele 2 sites in different cell types/donors? It seems that the ratio can have a wide range (Sup. Fig. 9a-c) in different cell types. When the ratio is near 0 or 1, it may become difficult to delineate the paternal vs the maternal cells. Have the investigators examined this systematically and had any advice in what biological context the approach is sufficiently powerful to be applied?

*We appreciate the reviewer’s insight on this topic. The ranges of XCI between the maternal and paternal allele has been examined in many papers and the overall skew is due to the low number of progenitor cells present during XCI, leading to biases that propagate during cell division. For our application, the most instructive result is by a study that surveyed 1,005 phenotypically normal females for XCI patterns. They concluded that 8.8 % of females in their cohort had an XCI skew greater than 80:20 or 20:80, 1.8 % 10:90/90:10, with a mean of 49:51 (paper:* [*https://doi.org/10.1086/507565*](https://doi.org/10.1086/507565)*). Therefore, it is widely applicable to cells from most female donors. We have emphasized this in the revised text:*

*Importantly, by analyzing X-linked variants of females included in the expanded 1000 genomes we found that the number of heterozygous SNPs found on the X chromosome in female humans is well above the required variation across the human population (****Fig. 1e****,* ***Supplementary Figure 3****). Moreover, a previous study found that only a very small fraction of the female population has a heavily skewed XCI ratio, with a mean ratio 48:52 in adult females^8^ Therefore, the XCI deconvolution approach is widely applicable to human cells from any female donor.*

*On the other hand, we found a difference in the XCI ratio within certain cell type clusters which we believe reflects clonal structures and clonal dominance. Some are clearly apparent due to existing clonal markers that we can measure. For example, the CD8+ T cells (EM)/CD4+ CTL cluster in donor 4 have the largest clones based on TCR sequence and they happen have the already dominant allele 2 active. In the CD8+ T cells (EM)/CD4+ CTL cluster in donor 7 we find the first and third largest clones based on the TCR, which have the (in this donor) less dominant allele 2 active. These two clones change the ratio to be close to 50:50 in this cluster in donor 7. We see a similar shift in the memory B cell cluster in donor 7. We note this in the revised text:*

*More than 99% of cells from the two female donors, Donor 4 and Donor 7, could be assigned an XCI status (****Fig. 2b****). Most cell clusters followed the XCI bias that was observed in the overall cell population, although a few cell clusters were inconsistent with the overall bias, suggesting exceptionally large clones present in those clusters (e.g. “CD8+ T cells (EM)/CD4+ CTL” in Donor 4, “CD8+ T cells (EM)/CD4+ CTL” and “Memory B cells” in Donor 7,* ***Supplementary Figure 4a).***

R1 Major Concern 4:

Improve writing and clarity:

The writing is a bit sketchy and there is a bit of disconnect from text to figures. For example,

- The manuscript states (Line 59 - 61), "Further visualization of the most abundant T cell clones and their respective XCI status showed that effector memory CD8+ T cell population shows the highest level of clonal expansion in both donors, and mucosal associated T cells in Donor 7 (Fig. 2d)." However, Figure 2d does not provide explicit labeling of cell types, making it challenging to verify the author's conclusions regarding clonal expansion in specific T cell populations. Clarification or additional labeling in Figure 2d is necessary for readers to accurately assess these findings.

*We thank the reviewer for their comment. We have revised Figure 2d to improve clarity.*

- The manuscript states (Line 103- 105), "Finally, we found that gamma-delta T cells do not share mutations with other T cell lineages, suggesting these subsets are produced by a separate set of progenitors than CD4+ and CD8+ T cells." However, similar observations of low relative covariance between these cell types are also evident in fate coupling analysis using other filtering criteria, as shown in Figures 2j and 2k. This indicates that the conclusion regarding gamma-delta T cells is not exclusive to the method presented in this study. It would be beneficial for the authors to acknowledge this and discuss how their method's findings align with or differ from these existing observations. Such a comparison would provide a clearer understanding of the novel contributions and potential limitations of the proposed method.

*We thank the reviewer for their comment and appreciate the need to transparently discuss the findings. A direct comparison of the Gamma-delta T cell clusters to the other T cell clusters show the impact on in the analysis our approach enable. Examining the profiles shows the clear difference between the normalized covariances of the different filtering criteria. Therefore, we think the conclusions are exclusive to the method we present.*

Reviewer Figure 1: Normalized covariance for Gamma-delta T cells compared to other cell clusters, from heatmaps in Figure2j-l.

*The text has updated to highlight this:*

*Finally, we found that gamma-delta T cells do not share mutations with other T cell lineages. Although the gamma-delta T cells clusters were coupled without XCI-informed selection of variants, they shared many mutations with other T cell lineages that were removed by the XCI-informed selection step, suggesting these subsets are produced by a separate set of thymic progenitors than CD4^+^ and CD8^+^ T cells.*

- Conclusions of Figure 2i (Line 98 to Line 103) about the lineage relationships and mutation sharing among T cell subsets are not directly interpretable from the figure. For example, the statement 'naïve CD4+ T cells were not coupled with a subset of CD4+ CM/EM T cells' (Line 100) appears to be contradicted by the high covariance observed between these two cell types in Figure 2i. This discrepancy between the text and the figure raises questions about the accuracy of the conclusions drawn.

*We thank the reviewer for catching this typo, we were referring to another cell cluster than the CD4+ CM/EM T cells cluster. We have revised the text to the following:*

*However, naïve CD4^+^ T cells were not coupled with a subset of CD4^+^ CM T cells.*

- The manuscript does not sufficiently contextualize these findings within the broader scope of existing research. A discussion comparing these observations with previous studies or established knowledge in the field is necessary. This would help in understanding how these new findings align with or challenge the current understanding of T cell lineage differentiation and development.

- Finally, the discussion should extend beyond the immediate results to explore the broader implications of these findings. How do they contribute to our understanding of T cell development and differentiation? Are there potential clinical or research applications that these findings could inform?

*We understand the need to discuss the findings within a broader scope, and have added this following text to provide the necessary context:*

*Several models have been proposed for T cell development, where e.g., the lineage relationship between memory and effector T cells remains unclear ^12^. Furthermore, while the gamma-delta subset of T cells is phenotypically different from alpha-beta T cells (CD4+ and CD8+), it is unclear which thymic progenitors produce gamma-delta T cells and the extent to which they share progenitors with alpha-beta T cells^13,14^. In mice, the timing alpha-beta and gamma-delta T cell fate decisions are quite well characterized^15^. However, the development of human gamma-delta T cells is less clear^16^. Understanding the origin of gamma-delta T cells may have implications for their role in tumor immune surveillance^17^.*

R1 Major Concern 5:

Improve Application Scope:

Can the approach be generalized to broader settings?

- The method's reliance on X chromosome inactivation (XCI) inherently limits its use to female samples. However, can the knowledge (e.g., mutation sites) learnt be generalized to male samples?

*We thank the reviewer for their comment. We appreciate the suggestion, and we investigated the extent to which we can generalize our finding to male samples but did not come to any conclusions within the scope of this study. As such, there are no additions to the manuscript regarding this question. However, we envision that upcoming studies may provide advances for this purpose, where our approach can be used for training a machine learning model with datasets obtained from female genomes to infer clonality in male datasets.*

- Can the approach be generalized to genomic RNA?

*We thank the reviewer for their suggestion to show the approach can be generalized to genomic single nucleotide variants. In this direction, we performed another analysis of the PBMC donor 7 dataset on nuclear SNVs detected by the recently published software Monopogen (10.1038/s41587-023-01873-x), which demonstrated a proof of principle that this approach can be applied to any somatic genomic variation. In particular, we have added this paragraph to the main text detailing the results and its accompanying figure panel:*

*Apart from the mitochondrial genome, the nuclear genome may contain somatic single nucleotide variants (SNVs) which are informative of clonal relationships. To demonstrate that our approach can be applied to nuclear SNVs, we applied the recently published variant calling software Monopogen to the Donor 7 PBMCs^22^. After the application of hard filtering steps used by Monopogen, we detected 1,153 putatively somatic nuclear SNVs. Most of these SNVs were detected in 2-10 cells, which is consistent with the clonal diversity in the PBMC dataset we analyzed (****Supplementary Figure 14a****). Considering XCI status of the cells harboring these putative SNVs, we filtered away 759 putative SNVs with inconsistent XCI status, with 394 mutations remaining (333 with allele 1 active and 61 with allele 2 active). The SNVs present in a relatively high number of cells all had inconsistent XCI status (****Supplementary Figure 14b****). The majority of these mutations were found in 2 cells, with a handful of mutations found in larger groups of cells (****Supplementary Figure 14c-e****). About 20% of cells had at least one clonal nuclear somatic mutation detected across cell clusters (****Supplementary Figure 14f****). These results demonstrate the use of XCI status to select clonally informative nuclear somatic SNVs, although further research is needed to account for the significantly lower variant coverage and variation in gene expression across cell types which may distort inferred lineage* *relationships*.

R1 Minor Concern 1:

Mislabeling of Figure Legends in Fig 2h and g: The legend descriptions for parts 'h' and 'g' appear to be swapped in relation to their corresponding plots. (Line 319 - 321)

*We thank the reviewer for catching this error. Fixed.*

R1 Minor Concern 2:

Clarification Needed for Figure 2d Labeling: In the manuscript, there is a reference to Figure 2d for the visualization of the most abundant T cell clones and their XCI status, specifically mentioning effector memory CD8+ T cells and mucosal-associated T cells in Donor 7 (Line 59 - 61). However, I noticed that the cell types are not explicitly labeled in Figure 2d. Please add clear labels to Figure 2d to accurately reflect the cell types being discussed.

*We thank the reviewer for their comment. As stated previously, we have revised Figure 2d to improve clarity.*

R1 Minor Concern 3:

Concerns on threshold selection in the binomial mixture model: In the manuscript, the threshold for mutation filtering in the binomial mixture model is set at 0.04 (Line 219), but the rationale behind this specific value is not clear. It would be beneficial for the author to provide justification for this choice, particularly considering its impact on the accuracy and reliability of the model in different datasets and biological contexts. An optimally set threshold is crucial to minimize the risk of false positives or negatives. Therefore, a more detailed justification or a method for determining this threshold in different research scenarios would be highly beneficial.

*The application of this threshold (0.04) was done due to the low sensitivity in detecting variant at lower heteroplasmy levels, both in terms of mutation refinement by XCI and mutation calling in individual cells. This decision is now supported by the comprehensive evaluations we have made because of your review.*

R1 Minor Concern 4:

Concerns Regarding the Claim of an "Unbiased" strategy: I would like to address the use of the term "unbiased" as mentioned in the Discussion section (line 138). In statistical inference, "unbiased" typically refers to methods or estimators that are demonstrably free from systematic error, meaning their expected value is equal to the parameter being estimated.

- For a claim of unbiasedness to be substantiated, it is generally expected that there be either a formal mathematical proof or a comprehensive set of simulation studies. These should demonstrate the estimator's unbiased nature across a variety of scenarios and parameter settings.

- As an alternative or in addition, a comparative analysis with other established methods, highlighting the relative absence of bias in the proposed method, would be valuable. This would provide a clearer understanding of the method's position in terms of bias compared to existing approaches.

*We thank the reviewer for their concern. To avoid any confusion, we have removed that word in our discussion since it was not our intention to imply the mathematical meaning of that word.*

Reviewer 2

In this work, authors show that by leveraging the information about the X chromosome inactivation, they get rid of some of the noise typical in somatic mutation calling and improve the analysis of mitochondrial mutations in single cells coming from females. The idea of identifying the X chromosome inactivation status is simple, but very useful. For example, to later identify the clonal evolution, the input signal from just one chromosome is cleaner.

*We thank the reviewer for their positive comments and interest in our work.*

R2 Major Concern 1:

The method the Authors use is not theirs. They use vireo https://eur01.safelinks.protection.outlook.com/?url=https%3A%2F%2Fgenomebiology.biomedcentral.com%2Farticles%2F10.1186%2Fs13059-019-1865-2&data=05%7C02%7Cjonas.frisen%40ki.se%7Cf3889e1e9c1f470c502308dc330b94d8%7Cbff7eef1cf4b4f32be3da1dda043c05d%7C0%7C0%7C638441372039450441%7CUnknown%7CTWFpbGZsb3d8eyJWIjoiMC4wLjAwMDAiLCJQIjoiV2luMzIiLCJBTiI6Ik1haWwiLCJXVCI6Mn0%3D%7C0%7C%7C%7C&sdata=lioVTfDMBNI8Sy1Wdt4ii4V7ndgUJ6ZZvAFWV9fbcrk%3D&reserved=0

Vireo is a powerful approach for identifying genotypes in single cell data and can be used for various applications, such as demultiplexing. However, this approach is not new, the method has been previously published and is widely used.

*We thank the reviewer for their comment. We agree with the reviewer that the main text was not sufficiently clear regarding the use of other tools. We have now revised the text in multiple places to clarify this:*

*We first sought to demonstrate how the XCI status of single female cells can be determined by using a Bayesian XCI deconvolution approach for predicting the active X chromosome in single cells from single-cell RNA sequencing (scRNA-seq) data (Methods)^6,7^. We utilize the demultiplexing tool Vireo to determine XCI status. By restricting the variants used for the deconvolution to the X chromosome and specifying two expected donors, the Vireo algorithm effectively detects the paternal and maternal allele.*

*We also clarify the use of mgatk:*

*We focused on Donor 7 due to the high mitochondrial genome coverage (106X,* ***Fig. 2f****), compared to Donor 4 (49X, data not shown). We obtained the initial set of mitochondrial mutations using the mgatk software, established quality filtering thresholds identified 766 putative somatic mitochondrial mutations (****Fig. 2g****), and after discarding mutations where neither allele passed the clonal heteroplasmy threshold, 689 mutations remained (Methods).*

R2 Major Concern 2:

On top of vireo, the authors use a binomial mixture model to call activated chromosome mutations. It is a very simplistic model. In particular, it accounts for the false positive error rate - the prob. that the mutation which is not present in reality, will be observed due to technical noise. In fact, the opposite may also happen (existing mutation will not be observed). In most generality, there are four different nucleotides that can be observed and only one of them is true. The authors do not consider that at all and should at least mention this as a limitation. On the side of the methodology, it is really nothing impressive.

*We thank the reviewer for their comment. In response to this concern and the concern of Reviewer 1, we have assessed the specificity and sensitivity of the mutation calling using the binomial mixture model. We discuss this explicitly in the main text:*

*Moreover, we also assessed the sensitivity and specificity of mutation calling in single cells using the binomial mixture model and we found excellent sensitivity at high specificity at heteroplasmy level 4% and above (****Supplementary Figure 6****).*

R2 Minor Concern:

The authors in their procedure use an arbitray cutoff of 0.04 for \theta_1 and \theta_2. Please add more clarification to motivate the use of this particular cutoff.

*We thank the reviewer for their comment. We selected the cutoff of 0.04 due to the high sensitivity in mutation calling for heteroplasmy levels above 4% at the read coverage levels we have in the analyzed data. We have now justified its use in the main text based on the above analysis and a simulation approach:*

*We assessed our approach to detect clonal mutations using a simulation approach which modelled many possible factors that might impact sensitivity of detection. The factors included XCI ratio, number of cells profiled, the fraction of cells in the population with the mutation, heteroplasmy level of the mutation, and the number of sequencing reads covering the variant. The main factors that impacted sensitivity were heteroplasmy level and read depth. XCI ratio did not have an impact on sensitivity. At the read depths commonly used for mitochondrial mutation discovery (100x coverage), our approach is generally able to detect clonal mutations at a heteroplasmy level of 4% and above (****Supplementary Figure 5****). Moreover, we also assessed the sensitivity and specificity of mutation calling in single cells using the binomial mixture model and we found excellent sensitivity at high specificity at heteroplasmy level 4% and above (****Supplementary Figure 6****).*

**2^nd^ round**

**Reviewer 2**

I thank the Authors for addressing my comments and also the comments of Reviewer 1

The review did not change the fact that the approach is from the computational perspective very basic and not really novel.

However, the method does seem to work well on the specific task that it is assigned, that is calling of mitochondrial mutations in single cells of females.

**Further advice from Reviewer 1:**

I think leveraging the available X inactivation status to refine mutation calling & clonal analysis is quite innovative. This practical approach could be integrated as a standard quality control check for **single-cell female samples**.  For the second point, I admit that the implementation is limited to female samples only
